# Targeted editing and evolution of engineered ribosomes in vivo by filtered editing

**DOI:** 10.1038/s41467-021-27836-x

**Published:** 2022-01-10

**Authors:** Felix Radford, Shane D. Elliott, Alanna Schepartz, Farren J. Isaacs

**Affiliations:** 1 Department of Molecular, Cellular, and Developmental Biology, Yale University, New Haven, CT 06520 USA; 2 Systems Biology Institute, Yale University, West Haven, CT 06516 USA; 3 Department of Chemistry, University of California, Berkeley, CA 94720 USA; 4 Department of Molecular and Cell Biology, University of California, Berkeley, CA 94720 USA; 5 Department of Biomedical Engineering, Yale University, New Haven, CT 06520 USA

**Keywords:** Synthetic biology, Ribosome

## Abstract

Genome editing technologies introduce targeted chromosomal modifications in organisms yet are constrained by the inability to selectively modify repetitive genetic elements. Here we describe filtered editing, a genome editing method that embeds group 1 self-splicing introns into repetitive genetic elements to construct unique genetic addresses that can be selectively modified. We introduce intron-containing ribosomes into the *E. coli* genome and perform targeted modifications of these ribosomes using CRISPR/Cas9 and multiplex automated genome engineering. Self-splicing of introns post-transcription yields scarless RNA molecules, generating a complex library of targeted combinatorial variants. We use filtered editing to co-evolve the 16S rRNA to tune the ribosome’s translational efficiency and the 23S rRNA to isolate antibiotic-resistant ribosome variants without interfering with native translation. This work sets the stage to engineer mutant ribosomes that polymerize abiological monomers with diverse chemistries and expands the scope of genome engineering for precise editing and evolution of repetitive DNA sequences.

## Abstract (web summary)

Genome editing methods are limited by the inability to selectively edit repetitive sequences. Here the authors demonstrate precise editing of a repetitive genetic element, a ribosome, while avoiding edits to native sites sharing identical sequence.

## Introduction

Genome editing introduces targeted modifications in the chromosomes of living cells, permitting the elucidation of causal links between genotype and phenotype, global reprogramming of cellular behavior, and emerging applications for gene therapy^1^. Nuclease-dependent approaches to genome engineering, such as CRISPR/Cas9, generate DNA double-stranded breaks (DSBs) to introduce modifications into the genome^2,3^. Such approaches are well-suited for gene disruption applications by non-homologous end joining and gene editing at single to few loci by homology-directed repair (HDR) across diverse organisms. Other approaches, such as prime editing^4^, base editing^5^, and multiplex automated genome engineering^6,7^ (MAGE), do not introduce DSBs for genome editing and can be employed for multisite genomic edits. However, a major limitation with all genome editing approaches is the inability to selectively edit or diversify repetitive genetic elements that possesses high sequence homology to other genetic loci (Supplementary Fig. 1), and which comprise large fractions of genomes across all domains of life.

The ribosome is one of the most conserved and structurally complex repetitive genetic elements found in nature, and one that is essential for the functioning of all organisms. In *Escherichia* *coli*, the ribosome is encoded by seven distinct genetic elements to support the essential function of protein synthesis. For decades, researchers have sought to mutate the ribosomal RNA to understand the impact modifications have on its structure and function. More recent efforts have used in vitro and directed evolution methods^8,9^ to generate mutant ribosomes to identify essential nucleotides (nt) responsible for tuning specificity to mRNAs^10,11^, modifications that confer resistance to antibiotics^12,13^, and engineered variants that accommodate abiological monomers in vitro^14,15^. An important advance towards ribosome modification was the Squires strain, in which all endogenous ribosomal operons were deleted, and a single *E. coli* rRNA operon was maintained on a multicopy plasmid^16^. Despite these advances, in vitro ribosome evolution methods have been limited by laborious serial manipulation of plasmids and cannot be used for parallel and continuous directed evolution of multiple translational components directly in cells. In vivo evolution efforts have been hampered by the need to isolate engineered ribosomes from their native context, such that they do not cross-react with native mRNAs^10,11,17^ or exchange mutant large and small subunits with native ribosomes. Mutagenesis of the anti-Shine-Dalgarno (aSD) site has generated a collection of ‘orthogonal’ ribosomes with limited ability to initiate translation of endogenous mRNAs in *E. coli*. Paired with ‘orthogonal’ mRNAs, these orthogonal ribosomes have advanced the development of isolated translation systems in cells. More recently, the development of orthogonal-tethered ribosomes (oRiboT^18,19^), which physically link the large and small subunits of the ribosome, prevent these mutant ribosomes from exchanging the large or small subunits with the native ribosomal subunits. This enables selective evolution of the tethered ribosome, due to functional insulation from the cell’s native translational apparatus^18,19^. The unresolved challenges in ribosome reengineering require more sophisticated approaches to edit and evolve engineered ribosomes at multiple sites (e.g., large and small subunits) inside cells alongside the native ribosome, which are encoded by multiple identical genes. In contrast to in vitro cloning and mutagenic technologies, genome editing methods could permit iterative edits at multiple sites and the generation of more complex ribosome libraries. More specifically, multiplex genome editing permits the evolution of multiple loci directly in the genome of cells and has been demonstrated to create mutagenic libraries with complexities that span 10^8^-10^9^ unique mutants^6,20^. To date, it has not been possible to use traditional genome editing approaches for the precise editing and molecular evolution of ribosomes in vivo due to extensive sequence homology with native ribosomal operons.

**Fig. 1:**
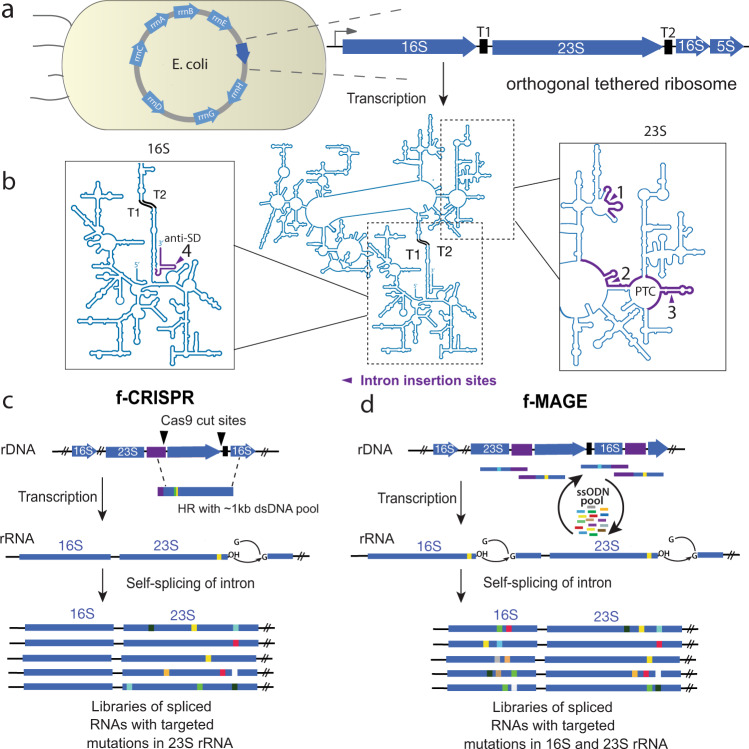
Filtered editing: methods to edit and diversify repetitive genetic elements. **a** There are seven native ribosomal operons (light blue) in the *E. coli* genome, which share extensive sequence homology to an orthogonal-tethered ribosome (oRiboT, dark blue), which is also introduced into the genome. **b** The secondary structure of oRiboT rRNA. Introns were introduced at four separate sites in oRiboT. Intron insertion sites are designated with purple arrows, and areas that can be targeted with filtered editing highlighted in purple. **c**, **d** In f-CRISPR and f-MAGE, an intron is introduced near the site targeted for mutagenesis to provide a unique address for hybridization of **c** sgRNA to introduce a Cas9-mediated double-stranded break, or **d** a pool of mutagenic MAGE ssODNs. After the mutation is introduced into the DNA, the intron is spliced out of the transcribed RNA to produce the desired product. In this work the *Tetrahymena thermophila* and a panel of other group 1 self-splicing introns were introduced into the gene encoding the orthogonal-tethered ribosome oRiboT to distinguish it from the seven native ribosome genes; f-CRISPR and f-MAGE were then used to generate libraries of spliced oRiboT RNAs with targeted mutations.

In this study, we describe ‘filtered editing’, to overcome limitations of previous genome engineering approaches and permit selective mutagenesis of repetitive genetic elements in cells. We inserted self-splicing introns into the rRNA gene encoding the oRiboT^18^ to break the sequence redundancy with the seven homologous rRNA genes native to *E. coli* (Fig. 1a, b) and act as uniquely addressable sites to direct genome edits to a desired locus, excluding all repetitive sequences in the genome. We reasoned that the placement of an intron within the oRiboT gene would establish a unique genetic address across the junction of the intron and repetitive genetic element (i.e., exon) that could be targeted for modification. We hypothesized that mutations could be introduced exclusively into the oRiboT gene using commonly used gene editing methods—CRISPR/Cas9^1,2^ (Fig. 1c) and MAGE^6^ (Fig. 1d) at one or multiple sites, differentiating it from the seven native ribosomal RNA genes. Self-splicing of the intron post-transcription would yield an rRNA containing targeted mutations. We demonstrated self-splicing of the intron and capability to perform selective editing with CRISPR/Cas9 and MAGE after integration of the oRiboT ribosome containing self-splicing introns into the chromosome of *E. coli* as well as on plasmid-borne constructs of oRiboT. We expanded this method to demonstrate utility with 10 additional self-splicing introns and the development of a novel engineered intron. These advances permitted the insertion of introns across multiple sites within oRiboT to enable multiplex filtered editing. We applied filtered editing to co-evolve the aSD sequence of the 16S rRNA to tune the ribosome’s translational efficiency and the peptidyl transferase center (PTC) and exit tunnel of the 23S rRNA to isolate mutant ribosomes containing new antibiotic-resistant mutations.

## Results

### The *Tetrahymena thermophila* intron is able to self-splice scarlessly while preserving ribosome function in vivo

**Fig. 2:**
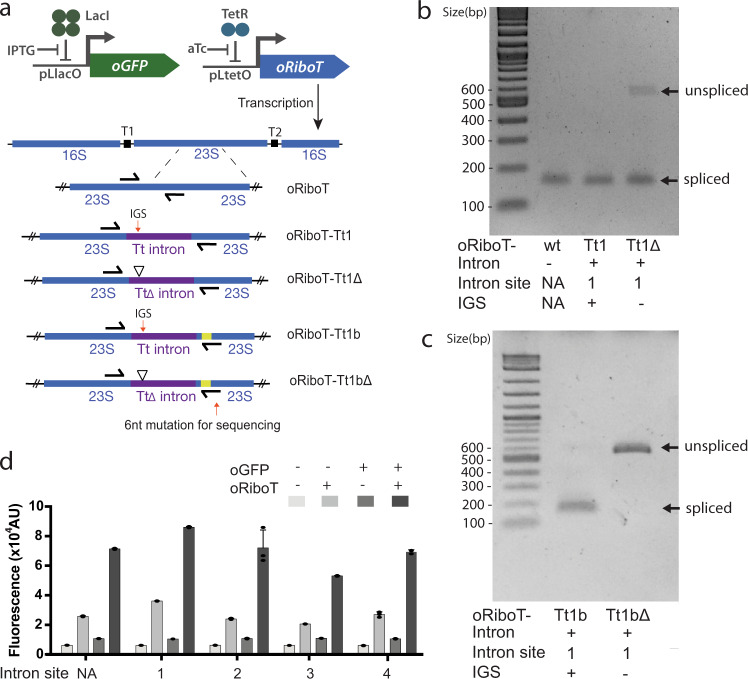
Validation of scarless intron splicing in vivo. **a** The oGFP reporter and oRiboT constructs used for oGFP expression and RT-PCR splicing validation experiments. The RNA constructs used to validate in vivo intron function by RT-PCR and verify the sequence of ligated exons are indicated below with functional or catalytically inactive (ΔIGS) introns indicated in purple. Sequencing primers are shown in black (Supplementary Table 3). The yellow band in oRiboT-Tt1b and oRiboT-Tt1bΔ indicates the site of a 6nt mutation inserted to distinguish oRiboT exon sequence from native rRNA, for absolute determination of splicing by RT-PCR. **b** RT-PCR was performed on total purified RNA from cells expressing oRiboT-Tt1 (+IGS) and oRiboT-Tt1Δ (ΔIGS) to validate self-splicing of introns in oRiboT. These are representative results from *n* = 2 independent experiments. **c** RT-PCR was performed on total purified RNA from cells expressing oRiboT-Tt1b (+IGS) and oRiboT-Tt1bΔ (ΔIGS) in order to determine whether introns were completely spliced out of oRiboT and ligated post-splicing. Both oRiboT-Tt1b and oRiboT-Tt1bΔ contained a 6nt mutation to distinguish them from native rRNA by RT-PCR. These are representative results from *n* = 2 independent experiments. **d** Expression of oGFP by WT oRiboT or variants whose genes contained Tt intron at sites 1, 2, 3, or 4. Values and error bars represent the mean and standard deviation of *n* = 3 biologically independent replicates.

We began by investigating whether the *Tetrahymena thermophila* type 1 self-splicing intron can be stably integrated and post-transcriptionally removed scarlessly to maintain the sequence and function of oRiboT^18^. The *Tetrahymena* intron was chosen because it is spliced naturally from the *T. thermophila* rRNA and functions effectively in both in vitro and in vivo contexts^21^. We inserted the intron into oRiboT immediately after position U1926 of the 23S rRNA (Site 1 in Fig. 1b, Supplementary Table 1), as this position represents the location of the intron within the *T. thermophila* ribosome, and has been previously demonstrated to function in *Escherichia coli*^22^. In order to obtain high rRNA yields specific to the oRiboT ribosome, we introduced a plasmid encoding oRiboT or oRiboT with the *T. thermophila* intron inserted (oRiboT-Tt1) into two independent C321.ΔA strains^23^ (Methods) of *E. coli*. We cultured these strains with oRiboT or oRiboT-Tt1 induction and performed RT-PCR on total purified RNA from each population using primers that amplified the region spanning the intron-exon junctions (Fig. 2a). A single band at 128 nt was observed in both cases (Fig. 2b), suggesting the complete and scarless splicing of the intron from oRiboT-Tt1. To confirm this finding, we created an oRiboT-Tt1 mutant (oRiboT-Tt1Δ) lacking the internal guide sequence (IGS) necessary for intron function (Fig. 2a). RT-PCR of total purified RNA from this strain revealed two bands, one at 128 nt indicating the presence of native ribosomes, and a second at 535 nt, indicating an unspliced product resulting from the IGS deletion (Fig. 2b). In this case, two bands were observed because the total purified RNA contained both the WT 23S sequence and oRiboT-Tt1Δ. In order to assess whether oRiboT-Tt1 was spliced correctly and to differentiate it from the cellular background, we used oRiboT-Tt1 and oRiboT-Tt1Δ as templates to construct two other mutants, oRiboT-Tt1b (+IGS) and oRiboT-Tt1bΔ (ΔIGS), which contain a unique sequence following the intron-exon splice junction (Supplementary Table 1, Fig. 2a) such that a primer can selectively amplify oRiboT-Tt1b or oRiboT-Tt1bΔ among the native ribosomes. Following RNA purification and RT-PCR from cell culture, we observed a single band at 128 nt for oRiboT-Tt1b, corresponding to the expected spliced product, and a single band at 535 nt for oRiboT-Tt1bΔ, corresponding to the expected unspliced product from the ΔIGS mutant (Fig. 2c). Finally, sequencing of oRiboT-Tt1b RT-PCR products confirmed the scarless ligation of the ribosome at the intron-exon junction post-splicing. Together, these results demonstrate stable integration of the *Tetrahymena* intron into the ribosome followed by scarless self-splicing from the oRiboT RNA precursor.

We next sought to determine the catalytic functionality of oRiboT ribosomes whose genes contain a stably integrated intron by assaying protein production in vivo. Since oRiboT contains an orthogonal anti-Shine Dalgarno sequence in its 16S rRNA^10,11^, we constructed a compatible GFP reporter plasmid (orthogonal GFP, oGFP) containing an orthogonal ribosome binding site sequence (oRBS)^11,18^ to establish an orthogonal ribosome—mRNA translation channel designed to limit cross-reactivity with native translational machinery (Supplementary Table 2). In this scheme, we expressed oGFP from the IPTG—LacI inducible P_L_-lacO promoter and plasmid-borne oRiboT from the aTc—TetR inducible P_L_-tetO promoter (Fig. 2a). We first assayed steady-state GFP expression in cells containing either oRiboT or oRiboT-Tt1 under all four induction conditions (−IPTG –aTc, +IPTG –aTc, −IPTG +aTc, +IPTG +aTc), and in each case observed equivalent GFP expression in cells containing oRiboT or oRiboT-Tt1 (Fig. 2d). Specifically, we observed the expected low levels of GFP without oGFP induction (−IPTG –aTc and –IPTG +aTc). Upon full induction (+IPTG +aTc), we observed high levels of GFP in cells containing either oRiboT or oRiboT-Tt1, demonstrating that the insertion of the *Tetrahymena* intron into the oRiboT-Tt1 ribosomal RNA and its subsequent removal does not interfere with protein translation. Notably, we also observed slightly elevated levels of oGFP fluorescence in cells induced with IPTG alone. Fluorescence was also verified in cells containing the oGFP construct alone (Supplementary Fig. 2), suggesting basal levels of translation from native ribosomes cross-reacting with oGFP mRNAs. This is consistent with previously reported characterization of tethered ribosomes^19,24^. This oGFP background is correlated with oRiboT function, thus as in oRiboT-Tt1 induced by aTc only (Fig. 2d).

### Precise editing of repetitive DNA sequences by filtered editing with CRISPR and MAGE

We next aimed to determine if the intron-ribosome junction could serve as a unique addressable site for targeted modification of oRiboT by CRISPR/Cas9^1,2^ and MAGE^6,25^. By using CRISPR/Cas9 to cut the intron-exon junction, along with applying a dsDNA pool harboring multiple desired mutations dispersed throughout a 1 kb region that targets the intron splice site (Fig. 1c), we hypothesized that mutations could be introduced exclusively into the oRiboT gene at one or multiple sites, differentiating it from other repetitive genetic elements. Similarly, by performing MAGE mutagenesis cycles with pools of mutation-bearing single-strand oligodeoxynucleotides (ssODNs) targeting the intron-oRiboT junction (Fig. 1d), mutations could be localized only to the oRiboT gene at the exclusion of other ribosomal targets. When the targeted oRiboT gene is transcribed, the intron is spliced out, releasing an rRNA containing targeted mutations.

**Fig. 3:**
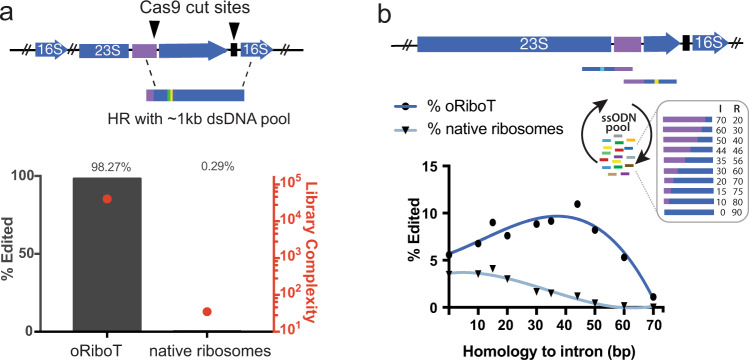
Characterization of f-MAGE and f-CRISPR performance. **a** f-CRISPR was performed on genomic oRiboT-Tt2 (Supplementary Fig. 3) to introduce dsDNA to replace a 832-bp region of oRiboT and introduce a 7 N mutagenic library; editing-efficiency was determined by deep sequencing. The left axis quantifies the percent of oRiboT or WT ribosomes that were edited (% edited). The red dot corresponds to the rightmost axis, which quantifies the library complexity. **b** f-MAGE was performed with ssODNs having 0–70 nucleotides of homology to the intron, in order to determine the optimum ssODN design parameters for targeted mutagenesis and reduction of off-target mutations. The percent of oRiboT or WT ribosomes that were edited (% edited) was determined with deep sequencing.

We designed a CRISPR array plasmid having two spacers with homology to the 5′ region of the intron, and to the linker between the 16S and 23S rRNAs that is unique to oRiboT, respectively. We then created a dsDNA containing 400 bp of homology directly upstream and downstream of the cut-sites and a 7-bp degenerate region to allow deep sequencing-analysis of both the allelic replacement frequency (ARF) and library complexity. We created a strain in which we genomically integrated pLtetO-oRiboT with *Tetrahymena* intron placed into site 2, with a distinguishing mutation for sequencing (oRiboT-Tt2-ed, Supplementary Table 1, Methods). We then introduced the CRISPR plasmid and dsDNA into the oRiboT-Tt2-ed strain, (Supplementary Fig. 3a). We grew cells to saturation and induced with Cas9 to select for cells with dsDNA replacing DSBs introduced into the *E. coli* chromosome. After isolating the genomic DNA of the selected cultures, we used paired-end next-generation sequencing (NGS) to quantify the ARF for oRiboT-Tt2-ed and WT ribosomes. We found extensive editing of oRiboT-Tt2-ed (98.27% ARF) and nearly undetectable levels of mutagenesis of WT ribosomes (0.29% ARF), even though the region mutagenized was identical to all seven native ribosomes (Fig. 3a, Supplementary Fig. 3b). Furthermore, we obtained 14,147 unique mutants out of a theoretical complexity of 16,384 (86.35% library coverage), demonstrating that complex libraries of mutants can be easily generated with this method while avoiding editing of unintended genomic sites sharing sequence similarity with the target locus.

We next sought to test this approach with MAGE, and assess the ability to introduce precise edits and generate diversity across multiple sites of oRiboT in vivo. MAGE introduces mutations using single-stranded DNA (ssDNA) oligodeoxynucleotides (ssODNs) that complex with ssDNA annealing proteins (*e.g*., λ Red Beta recombinase^26^) to hybridize to the lagging strand of a replicating chromosome^6,7^. Through cyclical introduction of complex pools of ssODNs, MAGE permits greater depth and breadth of mutation by avoiding toxicity associated with DNA DSB^6,7^. To first determine the optimal parameters for filtered-MAGE (f-MAGE), we designed ten 90-mer ssODNs to target the intron-ribosome junction. These ssODNs contained varying homology to the intron and exon and harbored mismatch mutations targeting the 23S oRiboT sequence of a chromosomally integrated oRiboT-Tt variant (Fig. 3b, Supplementary Fig. 4a). We performed one cycle of MAGE for each ssODN and then performed NGS to quantify the frequency of conversion at the oRiboT-Tt locus and at the seven wild-type ribosome genes. To restrict PCR-screening to only the oRiboT-Tt1 locus, we introduced a 22-nt mutation 108 nt upstream of the intron-exon junction (Supplementary Fig. 4a). ssODNs targeting exclusively the 23S rRNA sequence with no homology to the intron demonstrated ARFs of 4% and 5% at the native ribosomes and oRiboT-Tt1, respectively (Fig. 3b, Supplementary Fig. 4b,c). Notably, the measured ARFs at the native ribosomes represent a frequency shared across all seven sites, rendering the frequency of a mutation at any one of those sites <1%. As homology of the ssODN to the intron increased to 44 nt, we observed an increase in ARFs at the oRiboT-Tt1 locus to 11% coupled with a striking decrease at the native ribosome genes to <1% on average. As ssODN homology to the intron increased to 70 nt (20 nt homology to the ribosome), we observed almost undetectable (<0.0066%) ARFs at the native ribosomes with decreasing ARFs at oRiboT to 1.1%. Thus, optimal parameters for f-MAGE ssODN design^6,25^ may be context specific, in which 44 nt of homology to the intron (46 nt to the ribosome) maximizes conversion of oRiboT whereas 70 nt of homology to the intron (20 nt to the ribosome) renders off-target conversions at the seven native ribosome loci effectively undetectable (Fig. 3b, Supplementary Fig. 4c).

### Application of filtered editing for evolution of new ribosomes resistant to antibiotics

To demonstrate the utility of selectively evolving ribosomes with new function by f-MAGE while preserving native ribosomes in the same cell, we tested whether we could introduce targeted diversity within the exit tunnel to confer resistance to ribosome-targeting antibiotics. We used f-MAGE to reconstruct G2032A, G2057A, and A2058G mutations in both oRiboT and untethered ribosomes because these mutations have previously been shown to confer resistance to erythromycin, clindamycin, chloramphenicol, and lincomycin^27,28^ (Table 1). We expressed the rRNA from the strong inducible PL-tetO promoter on plasmids to enhance ribosome expression. We found that ribosomes with mutations generated by f-MAGE recapitulate these phenotypes, demonstrating increased growth in the presence of otherwise toxic concentrations of these antibiotics. We also observed growth in clindamycin for the G2057A WT mutant, despite this not being previously reported^27,28^. Interestingly, we observed differences in antibiotic sensitivities in RiboT-Tt2 (e.g., resistance to erythromycin in native RiboT-Tt2 variant), suggesting that the presence of the tether may cause functional changes in the ribosome that are not completely understood. (Table 1, Supplementary Note 1, Supplementary Figs. 6–9, top panels). Furthermore, while the ribosomes with mutations generated by f-MAGE all recapitulated previously reported phenotypes, the degree of growth in some cases was mild (e.g., growth of the A2058G WT mutant in lincomycin) (Supplementary Fig. 9).

**Table 1:**
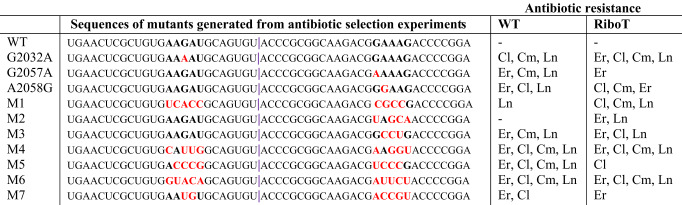
Mutants generated with f-MAGE having resistance to a panel of antibiotics.

23S RNA mutations both previously known and identified in this study, and their effects on antibiotic resistance in an oRiboT and WT ribosome background. The purple bar indicates the location of the Tt intron. Region mutagenized is indicated in bold. Mutations are indicated in red.

*Er* erythromycin, *Cl* clindamycin, *Cm* chloramphenicol, *Ln* lincomycin.

Motivated by these results, we then generated a complex library of RiboT-Tt2 ribosomes in order to discover new mutations that confer antibiotic resistance, and potentially have improved antibiotic resistance phenotypes compared to previously reported mutants (Supplementary Fig. 5). Seven new mutant ribosomes were identified that showed varying degrees of resistance to multiple antibiotics (Table 1, Supplementary Note 1). We re-transformed the plasmids containing the mutant ribosomes into a clean MG1655 genetic background and found that each mutant conferred resistance to a subset of the antibiotics (Supplementary Figs. 6–10).

To further validate these RiboT antibiotic-resistant mutants and assess the impact of the tethered linker, we reconstructed these mutations in the natural ribosome with the intron at site 2 (WT-Tt2). Most mutations mapped qualitatively to WT-Tt2, exhibiting a slightly weaker resistance phenotype, likely due to the higher basal resistance to antibiotics observed in RiboT-Tt2 (Supplementary Figs. 6–10). However, the antibiotic resistance of several mutants differed between the WT-Tt2 ribosome and RiboT-Tt2 (e.g., M2 for erythromycin and lincomycin, M3 for chloramphenicol, and M6 for clindamycin). Collectively, we identified seven new ribosome mutations that can be used to confer conditional orthogonality to native ribosomes or RiboT, and also reveals functional differences in the RiboT due to its tether, which warrants further investigation in future structural and functional studies.

### Bioprospecting and engineering group I introns for multiplex filtered editing

We next conducted two main experiments to assess whether a self-splicing intron can be introduced across multiple sites of the ribosome simultaneously to enable multisite filtered editing. First, we cloned ribosome variants in which the *Tetrahymena* intron was introduced across three additional sites within the large and small subunits (Fig. 1b). We demonstrated that the ribosome supported robust expression of oGFP, and that rRNA was scarlessly re-ligated post-splicing at each site (Supplementary Note 2, Fig. 2e). These results demonstrate that we can place introns into the ribosome across multiple sites, establishing unique genetic addresses for filtered editing across multiple ribosomal positions.

**Fig. 4:**
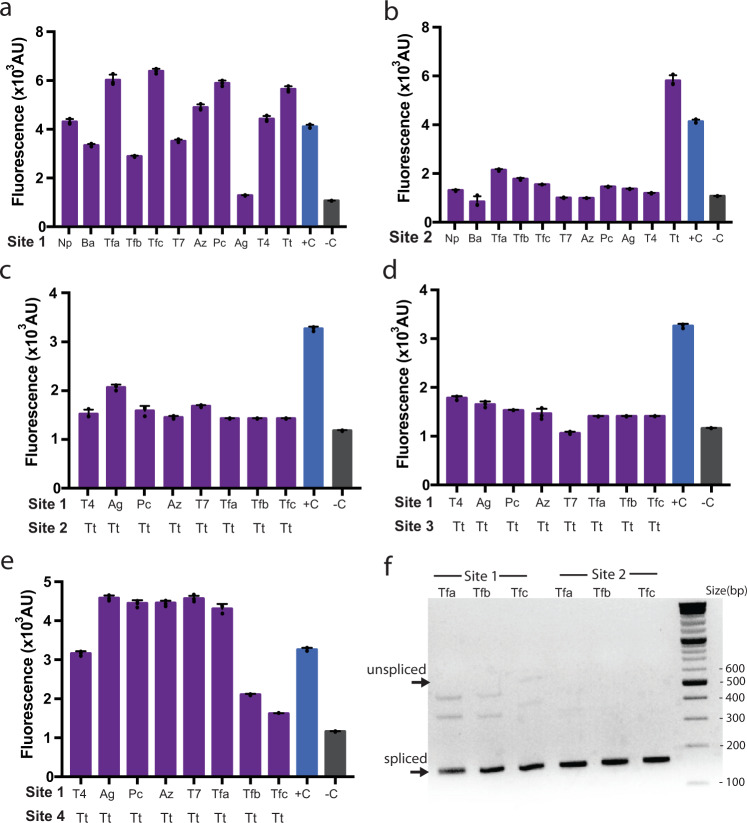
Characterization of intron library across multiple sites in oRiboT. **a**, **b** Additional group I self-splicing introns were tested for expression of oGFP when introduced into oRiboT at sites 1 or 2. **c**–**e** Fluorescence of oGFP-oRiboT variants with intron candidates at site 1 and *Tetrahymena* intron at site 2, 3, or 4. **f** RT-PCR was performed on total purified RNA from cells expressing oRiboT with Tfa, Tfb, or Tfc intron at site 1 or site 2 to determine whether introns were completely spliced out of oRiboT and ligated post-splicing. Intron abbreviations correspond to Supplementary Table 5; +C = oRiboT with no intron; −C = no oRiboT plasmid. Values and error bars represent the mean and standard deviation of *n* = 3 biologically independent replicates.

In a second set of experiments, we performed bioprospecting to identify ten Group I self-splicing introns and investigated whether these additional intron variants could serve as unique addressable sites for multiplex editing of two or more repetitive sites. We built and tested oRiboT variants with self-splicing intron candidates at sites 1 or 2, respectively (Fig. 4a, b, Supplementary Table 5). In selecting the introns, we employed a combined bioinformatics and literature-based approach to select group I introns that had been previously shown to self-splice in vitro or in vivo (Supplementary Table 5). We found that all introns tested were functional at site 1 (Fig. 4a) but showed reduced function at site 2 (Fig. 4b), in contrast to the *Tetrahymena* intron, which was functional at all sites tested. Nevertheless, several introns at site 1 demonstrated WT-levels of oRiboT function, suggesting that the use of these introns at site 1 in parallel with *Tetrahymena* at another site could enable editing at multiple loci simultaneously. To test this hypothesis, the introns that demonstrated the best performance at site 1 were subsequently used to construct oRiboT constructs having the new intron at site 1, and *Tetrahymena* intron at sites 2, 3, or 4, respectively. We found that all of the ribosomal constructs had robust activity with the *Tetrahymena* intron at site 4 and intron candidates at site 1 (Fig. 4e). However, introduction of the *Tetrahymena* intron at sites 2 or 3 ablated ribosomal function (Fig. 4c, d). To further validate splicing of these new intron candidates as we had done for oRiboT genes containing a *Tetrahymena* intron, we grew *E. coli* containing oRiboT with Tfa, Tfb, or Tfc introns at either site 1 or 2, and performed RT-PCR on total purified RNA from each population using primers that amplified the region spanning the intron-exon junctions (Fig. 4f). All of the introns showed splicing at both sites 1 and 2, yet ribosomes with newly-tested introns at site 2 showed reduced function. We hypothesized that the highly complex 3-dimensional structure of the ribosome may be a steric barrier to simultaneous splicing and ribosome assembly in some ribosomal locations (e.g., site 2). The additional space provided by the intersubunit bridge at site 1 could act to relieve this inhibition, allowing for more efficient intron splicing and simultaneous ribosome folding. The investigation of this mechanism could be a subject of interest in future work.

### Engineering chimeric introns for multisite filtered editing of ribosomes

**Fig. 5:**
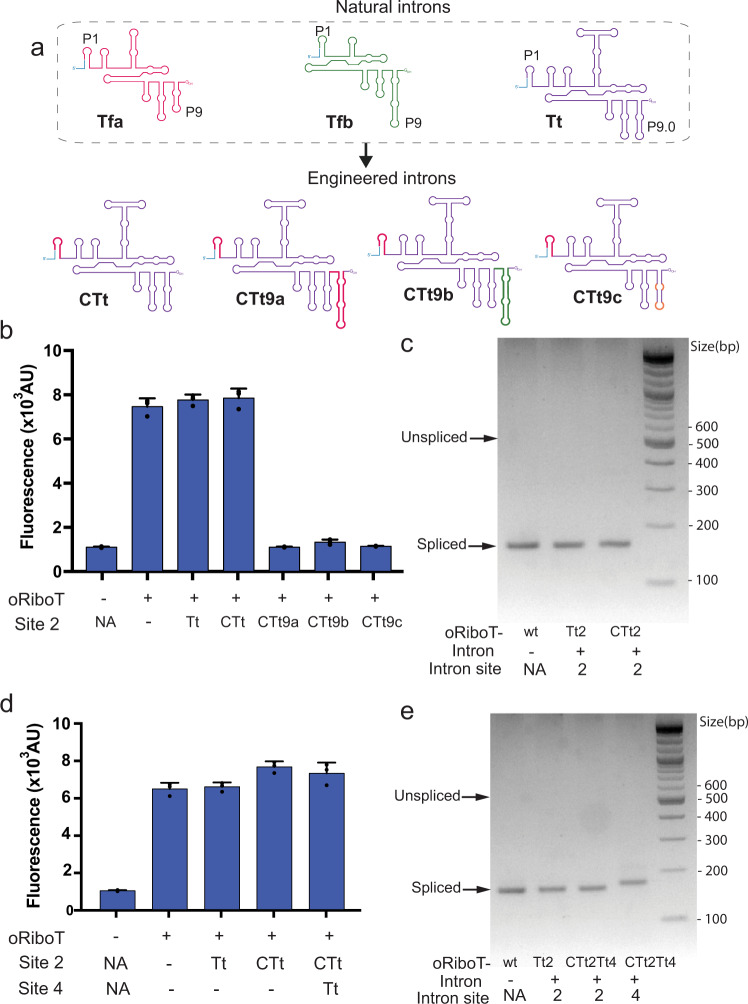
Engineering chimeric introns for multisite filtered editing. Chimeric introns were constructed by combining the P1 or P9 helices of the Tfa introns with the Tt intron, in order to generate an intron with unique 5′ and 3′ sequence. **a** The P1 helix of Tt was replaced with P1 from Tfa to produce CTt. CTt was further modified to P9 from Tfa or Tfb to produce CTt9a or CTt9b, respectively. CTt9c was created by altering the native sequence of bases in the *Tetrahymena* P9.2 in CTt while maintaining homology across the stem loop. **b** Expression of oGFP by WT oRiboT or variants whose genes contained Tt, CTt, CTt9a, CTt9b, or CTt9c intron at site 2. Values and error bars represent the mean and standard deviation of *n* = 3 biologically independent replicates. **c** RT-PCR was performed on rRNA to determine whether the CTt intron was completely spliced out of oRiboT. These are representative results from n = 2 independent experiments. **d** Expression of oGFP by WT oRiboT or variants whose genes contained introns at site 2(oRiboT-Tt1 or oRiboT-CTt2) or sites 2 and 4 simultaneously (oRiboT-CTt2Tt4). Values and error bars represent the mean and standard deviation of *n* = 3 biologically independent replicates. **e** RT-PCR was performed on rRNA to determine whether the Tt and CTt introns were completely spliced out of oRiboT when simultaneously present at sites 2 and 4, respectively. These are representative results from *n* = 2 independent experiments.

Having demonstrated the ability to introduce distinct introns across multiple sites in the ribosome and maintain ribosomal function, we next sought to expand filtered editing to introduce targeted modifications across multiple ribosomal sites in parallel. We hypothesized that since all introns we tested were group I introns, we could create chimeric introns by interchanging homologous elements between introns (Fig. 5a). We chose to develop variants of the *Tetrahymena* intron with the P1 helix and P9 helix replaced with homologs from the other introns tested in this study (Fig. 5a, Supplementary Note 3). Changes to P1 have been shown to be deleterious to *Tetrahymena* function^29^, but we hypothesized that P1 transplanted from introns demonstrating self-splicing activity could functionally compensate for the loss of the native P1 in *Tetrahymena*. To test this hypothesis, we created an oRiboT-variant with chimeric *Tetrahymena* introduced into site 2, containing P1 from Tfa (oRiboT-CTt2) (Fig. 5a). Upon assaying oGFP expression from oRiboT-CTt2, oGFP signal was comparable to WT oRiboT or oRiboT-Tt2 (Fig. 5b). Analysis by RT-PCR and subsequent sequencing of total purified RNA from oRiboT-CTt2 indicated complete and scarless splicing of the engineered intron from oRiboT-CTt2, just as the natural intron was spliced from oRiboT-Tt2. (Fig. 5c). This indicates that the ribosome assembled correctly and is functional after intron splicing. We also constructed three other intron variants, having variation in the P9 helix (CTt9a, CTt9b, CTt9c) (Fig. 5a, Supplementary Note 3), resulting in lower oGFP expression. Since the CTt intron was the only engineered intron that produced WT-level RiboT function (Fig. 5b), we proceeded with the CTt intron to apply filtered editing across two loci simultaneously.

### Multi-functional engineering of orthogonal ribosomes by multiplex editing and evolution

We next applied filtered editing to enable continuous multisite evolution of ribosomes in vivo. We chose to position the CTt intron at site 2, at the junction of the PTC/exit tunnel, and the Tt intron near the aSD Sequence (aSD) in the 16 S rRNA (site 4) (Fig. 1b) to co-evolve the large subunit and orthogonality of the aSD in the small subunit simultaneously (Supplementary Fig. 11a). To first validate whether a ribosome containing such a placement of introns would be functional, we constructed oRiboT-CTt2-Tt4 (Supplementary Note 3). We confirmed that oRiboT-CTt2-Tt4 signal was comparable to WT oRiboT (Fig. 5d), suggesting efficient self-splicing of two introns simultaneously and proper ribosome assembly. RT-PCR and subsequent sequencing on both intron positions within oRiboT-CTt2-Tt4 indicated complete splicing at both sites 2 and 4 and seamless ligation of the exon (Supplementary Note 3). A single band (~128 nt) was observed for both site 2 and 4 introns, with no evidence of the unspliced (535 nt) product (Fig. 5e).

Having validated an oRiboT construct with two mutually-orthogonal introns, we next sought to perform f-MAGE to validate in vivo editing at the aSD (site 4). We targeted site 4 of oRiboT-CTt2-Tt4 with an ssODN that would switch the orthogonal aSD to the WT *E. coli* aSD sequence. We performed six cycles of MAGE and then induced the cells for oGFP expression and visualized the cell populations from each MAGE cycle with flow cytometry (Supplementary Fig. 11). The fluorescence after each cycle shifted noticeably, and there was a dramatic enrichment of non-fluorescent cells observed after cycle 6 (80% non-fluorescent) (Supplementary Fig. 11c), suggesting that the aSD of most ribosomes in the population were mutated to WT and thus prevented oGFP translation. Sequencing of 10 clones plated from the sixth f-MAGE cycle confirmed that they all contained the WT aSD sequence. These results demonstrate the ability to selectively evolve orthogonal ribosomes in vivo with f-MAGE.

**Fig. 6:**
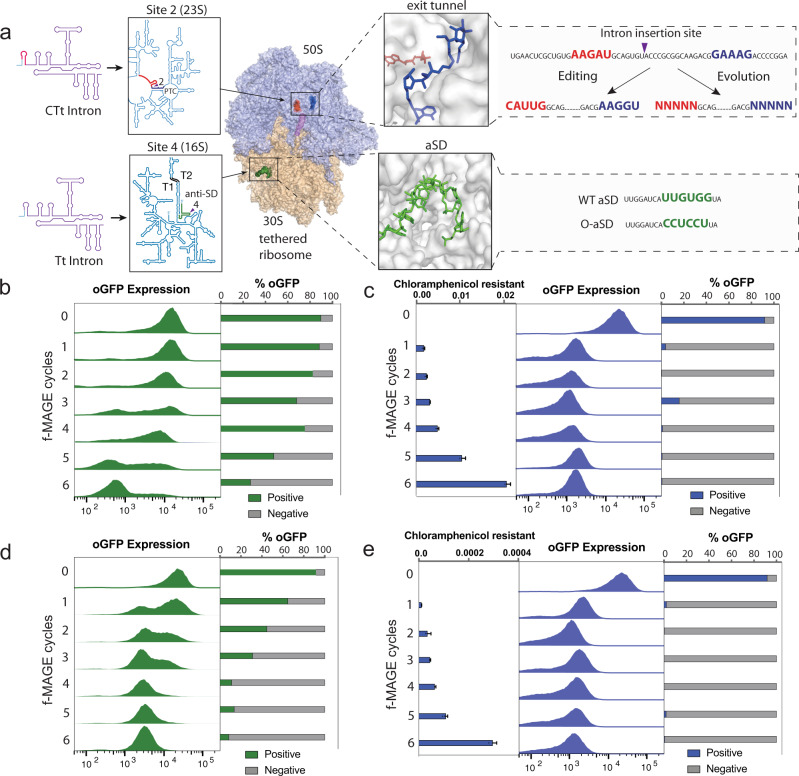
Applying f-MAGE to evolve antibiotic resistance and orthogonality in tethered ribosomes containing two introns. **a** Introns were introduced at two sites in oRiboT: the engineered (CTt) intron at site 2 and the natural Tt intron at site 4. These orthogonal introns enable mutagenesis of two subunits of the ribosome at three sites: exit channel (site 2), PTC (site 2), and anti-SD (site 4). **b**, **c** Continuous in vivo editing with f-MAGE was used to introduce antibiotic resistance at site 2 with defined M4 mutation (Table 1) and orthogonality in the small subunit at site 4. **b** Post-MAGE cultures were induced for oGFP expression and flow cytometry was performed on cultures from f-MAGE cycles 0–6. Percentage of oGFP-positive cells was quantified for all cycles, and **c** normalized CFU counts in chloramphenicol (7.74 µM). Chloramphenicol-selected cells were induced for oGFP expression and flow cytometry was performed. Percentage of oGFP-positive cells was quantified for all cycles. Values and error bars represent the mean and standard deviation of *n* = 3 biologically independent replicates. **d**, **e** Continuous in vivo editing with f-MAGE was used to evolve chloramphenicol resistance at site 2 and orthogonality in the small subunit at site 4. **d** Post-MAGE cultures were induced for oGFP expression and flow cytometry was performed on cultures from f-MAGE cycles 0–6. Percentage of oGFP-positive cells was quantified for all cycles, and (**e**) normalized CFU counts in chloramphenicol (7.74 µM). Chloramphenicol-selected cells were induced for oGFP expression and flow cytometry was performed. Percentage of oGFP-positive cells was quantified for all cycles. Values and error bars represent the mean and standard deviation of *n* = 3 biologically independent replicates.

We next sought to perform multisite editing of the large and small subunits in the same ribosome to evolve antibiotic resistance and tune orthogonality to mRNA in vivo. We performed 6 cycles of f-MAGE with a pool of two ssODNs targeting the exit tunnel (site 2) of the 23S rRNA and encoding the M4 mutation we previously identified to confer chloramphenicol resistance (Table 1), along with one ssODN editing the anti-oSD (site 4) of the 16 S rRNA of oRiboT-CTt2-Tt4. (Fig. 6a). We quantified the oGFP fluorescence with flow cytometry after each cycle of f-MAGE, performed growth curves in chloramphenicol, and measured CFUs on chloramphenicol plates. We observed a decrease in fluorescence with each cycle as the ribosome was edited from an orthogonal to a consensus aSD sequence (Fig. 6b). We also observed a gradual increase in growth in liquid media (Supplementary Fig. 12) and CFUs with successive cycles of f-MAGE (no growth without f-MAGE cycling and peaking at 0.02 survival ratio at cycle 6) (Fig. 6c). Furthermore, the ability to edit both the large and small subunits simultaneously enabled the evolution of functionally co-dependent phenotypes (e.g., an oRiboT AND gate): the modification of the aSD of oRiboT to be non-orthogonal and the introduction of mutations to the exit tunnel were both necessary for cell survival in chloramphenicol.

To evolve complex populations continuously across multiple ribosome sites, we performed f-MAGE in a strain containing oRiboT-CTt2-Tt4 with degenerate ssODNs targeting 5′ and 3′ of the site 2 chimeric intron. We created a diverse population (1.04 × 10^6^ theoretical complexity) with the aim of evolving chloramphenicol resistance while simultaneously tuning the orthogonality of this population via an ssODN that converts the orthogonal aSD to the consensus *E. coli* aSD sequence at site 4 (Fig. 6d). As before, we observed a decrease in fluorescence with each cycle until the population became non-fluorescent at cycle 6. This observation coincided with an increase in growth in chloramphenicol in liquid (Supplementary Fig. 13) and on solid medium (Fig. 6e). The ratio of surviving mutants increased dramatically after cycle 4 (Fig. 6e) as in the discrete editing experiment (Fig. 6c). The survival rate at cycle 6 using a degenerate pool of ssODNs (1.04 × 10^6^ theoretical complexity) was 3 × 10^-4^ (Fig. 6e), which was ~10^2^ lower than editing observed with a discrete oligo at site 2 (2 × 10^-2^) (Fig. 6e). This would be expected, given that more mutations in the degenerate ssODN population do not confer chloramphenicol resistance compared to the targeted M4 mutation in the discrete experiment. The survival of a population member depends on both conversion of the anti-SD to WT (site 4) and a mutation at site 2, which confers chloramphenicol resistance. In both the discrete and degenerate editing experiments, this interdependence would explain the delay in survival ratio observed until cycle 4, as both sites 2 and 4 would need to be edited in a single ribosome to ensure cell survival in chloramphenicol.

Induction of oGFP and the RiboT ribosomes of the selected populations enriched for non-orthogonal ribosomes (Fig. 6c, Fig. 6e: panels 2 and 3) as evidence by the loss of fluorescence in each population. This result is consistent with the RiboT mutants, containing chloramphenicol-resistant mutations near site 2, needing to be non-orthogonal to support cell survival under chloramphenicol selection. To further validate the ability to dissect unique phenotypes via continuous f-MAGE editing or evolution, we employed FACS to sort populations induced for oGFP expression from f-MAGE cycles 0–6 (Fig. 6b, d) into negative and positive bins and plated them to obtain CFU counts in chloramphenicol. We observed consistent survival of all cycles sorted for low fluorescence, and no growth for cells from the high bin, except for a trace amount of CFUs for cycle 6 (0.37% survival ratio compared to positive sorted bin), suggesting the emergence of rare escape mutants. Collectively, these data demonstrate the ability to co-evolve two functions simultaneously. Furthermore, filtered editing permits the dissection of phenotypic differences within complex populations of a repetitive genetic element, as a result of both discrete edits or the evolution of a complex population.

## Discussion

This study demonstrates a new method—filtered editing—that permits targeted genome editing and evolution of select repetitive genetic elements while preserving the integrity of native loci containing identical sequences. We demonstrated the utility of f-CRISPR and f-MAGE through targeted editing of the ribosome—one of the largest and most complex noncoding RNAs in the cell. Filtered editing permits the generation of a ribosome library across a cell population without the need for laborious plasmid cloning, site-directed mutagenesis, or re-transformations. This work allowed us to isolate new ribosome variants resistant to antibiotics, possessing tunable affinity to the RBS of mRNAs, and identify subtle impacts that tethered subunits have on ribosome function^19^. The precision afforded by filtered editing in both type and location of edits, as well as the targeting of only one repetitive genetic element out of multiple identical genomic sequences, distinguishes this method from previous evolution^30,31^ or genome engineering^2–6^ approaches. In prior work, only in vitro mutagenesis techniques could be used to evolve repetitive genetic elements such as ribosomes, at the cost of library complexity, and inability to edit native loci (Supplementary Table 6). Genome editing methods, while allowing more complex libraries, could not be used as they would edit all instances of a repetitive genetic element throughout the genome (Supplementary Table 6). Filtered editing permits the co-evolution of multiple, distal sites of a single repetitive genetic element directly in the genome. This allows for iterative introduction of precise edits that drive continuous evolution of dynamic genotypic diversity, while leaving the remainder of the cell’s genome unperturbed. Such capabilities hold promise for current challenges in synthetic biology, such as the systematic repurposing of the cell’s translational apparatus, which spans multiple components (e.g., tRNAs, aaRS, EF-Tu, and the ribosome).

The strategy we employed to perform multisite editing of the ribosome demonstrates the ability of filtered editing to introduce discrete edits and to generate targeted diversity in vivo. Self-splicing of the Tt intron was demonstrated at all four ribosome sites tested. Although the placement of two Tt introns in close proximity (i.e., sites 1 and 2) ablated ribosomal function (Fig. 4), bioprospecting efforts increased the number of functional self-splicing introns for filtered editing. We hypothesize that variable editing of these ten introns across the four ribosome sites tested could be explained by the slower catalytic rate of some of these introns^32,33^ compared to the Tt intron, presenting a barrier for simultaneous splicing of introns that preserve natural ribosomal folding and function. We overcame this constraint through the engineering of novel chimeric introns, one of which demonstrated scarless self-splicing and multiplex editing and evolution of the ribosome’s small and large subunits. We hypothesize that the additional space provided by the intersubunit bridge^34,35^ at site 1 could act to relieve kinetic barriers to simultaneous intron splicing and ribosomal folding arising because of steric crowding. In other noncoding RNAs that are structurally less complex than ribosomes, we expect the locations for intron insertion will be more permissive, allowing the broader application of this technique.

This study demonstrated the evolution of two distinct phenotypes with convergent functions in the ribosome, simultaneously editing orthogonality of oRiboT to mRNAs and its resistance to diverse antibiotics. Such co-evolution strategies can be expanded for evolving ribosomes and other translational components, such as aaRSs and tRNAs, in vivo, to enable systems-level engineering of the entire translation apparatus. For instance, the ability to evolve ribosomes with filtered editing can aid other efforts to engineer orthogonal ribosomes for the metabolic insulation of the translation of protein biopolymers, toxic enzymes, or metabolites^17,36^. We also anticipate that filtered editing could enable the continuous evolution of next-generation ribosomes able to polymerize abiological monomers with diverse chemical structures^15,37–39^. Our work demonstrated the evolution of an exogenous tethered ribosome (oRiboT), and we anticipate that filtered editing can be adapted to also edit native ribosomal operons in their genomic contexts. For example, all *E. coli* ribosomes have unique sequence context 5’ and 3’ of each ribosomal operon. Indeed, this has been recently used to delete all native ribosomal operons and replace them with their engineered counterparts^19,40^. While deletion or replacement of individual ribosomal operons in the genome has been demonstrated, it has not been possible to precisely and continuously edit a repetitive genetic element such as a ribosome directly in the genome. We anticipate filtered editing could open possibilities for such studies.

Adapting filtered editing to both CRISPR/Cas9 and MAGE genome editing methods establishes broad utility and flexibility. While f-MAGE can be used to introduce deep edits near a chosen intron, f-CRISPR can expand the space over which mutations can be introduced, generating distributed edits between two introns at a large distance (>1 kb). This feature is ideal for evolving complex populations for desired phenotypes where the mutagenic landscape of the population can be continuously refined and assayed in vivo. Given the species-independence of both CRISPR/Cas and the Group I introns characterized in this study, we anticipate that f-CRISPR can be ported to other bacteria or eukaryotes to edit and evolve the ribosome or other repetitive genetic elements (e.g., tRNAs, ncRNAs, or transposable elements), which are ubiquitous across all domains of life and, for example, comprise 50–70% of the human genome^41,42^. Selective modification of repetitive genetic elements would permit their functional characterization and establish new avenues to alter cellular physiology. Specifically, deletion of transposable elements enhances genome stability^43,44^, mutagenesis of CRISPR arrays affects innate immunity^45^ and genome editing^46^, and translational components (*e.g*., tRNAs, aaRS, ribosomes) can be co-evolved for genetic code expansion^47^.

## Methods

### Strains and culture conditions

Two strains of *E. coli*, the wild-type MG1655 (CP027060.1, GI:1352181442) and GRO C321.ΔA (CP006698.1, GI:54981157)^14,37,38^ that lack all TAG codons and release factor 1, were used and modified in this study. The C321.ΔA strain is derived from strain EcNR2 (Δ*mutS*:*cat*Δ(*ybhB*-*bioAB*):[*cI857*Δ(*cro-ea59*):*tetR-bla*]), modified from *E. coli K-12 substr. MG1655* as previously described^27,28^. The original C321.ΔA was modified by replacing the carbenicillin gene with spectinomycin using standard λ-Red recombination^14,37,38^ to permit compatibility with plasmid constructs containing the engineered ribosomes. C321.ΔA_spec was grown in low salt LB-min medium (10 g tryptone, 5 g yeast extract, 5 g NaCl in 1 L dH_2_O) at 34 °C. Variants of this strain were constructed to contain the chromosomal- and plasmid-based oRiboT and wild-type ribosome constructs described below. All strain variants were grown under the same conditions with the exception of supplementation with inducers (aTc, IPTG) or antibiotics as described.

### Plasmid construction

Plasmids containing the tethered ribosome (RiboT, pRiboT), the orthogonal-tethered ribosome (oRiboT, poRiboT), and oGFP (poGFP) were a kind gift from the laboratory of M. Jewett^48,49^. The promoters driving the expression of RiboT and oRiboT were replaced with PL-tetO^23^ such that transcription of the ribosome variants can be controlled by the TetR protein and anhydrotetracycline (aTc, Sigma). These plasmids were used as templates to construct all ribosome variants (Supplementary Table 1) described in this study.

Plasmids containing oRiboT with intron variants were constructed by insertion of gblocks (Integrated DNA Technologies) of introns with modified IGS (to match complementary site next to the 5’ intron start site) at sites 1 (after U1926, IGS = gggacc), 2 (after U2041, IGS = gcactg), 3 (after U2489, IGS = gccgcc), and 4 (after 16 S U1522, IGS = gggttc) into the oRiboT plasmid harboring a colE1 origin of replication and carbenicillin resistance marker. All plasmids were assembled using Gibson assembly (NEB). oRiboT-Tt4 and oRiboT-Tt4∆ were constructed by amplifying oRiboT-Tt1 and oRiboT-Tt1Δ plasmids, respectively, with primers containing desired mutations and assembled using Gibson Assembly.

### Genomic integration of oRiboT and variants

Clonetegration^50^ was used to introduce oRiboT-Tt1 into the genome of C321.ΔA_spec. pOSIP-CH (Addgene plasmid # 45980) and pE-FLP (Addgene plasmid # 45978) were gifts from Drew Endy & Keith Shearwin. Briefly, oRiboT-Tt1 was amplified by PCR and cloned into pOSIP-CH plasmid using Gibson assembly. After electroporation and overnight recovery, cells were plated on chloramphenicol plates. Following overnight growth, correct integration was verified by colony PCR. The attP sites of the correct integrants were then removed with MAGE in order to prevent re-excision. Following PCR and sequencing verification, pE-FLP (flipase), containing a heat-sensitive origin of replication, was transformed and used to remove the FLP-excisable integration module. Then pE-FLP was removed with overnight growth at 37^o^C.

### Filtered-CRISPR (f-CRISPR)

A strain with genomically integrated oRiboT-Tt2 with distinguishing mutation upstream of intron for NGS-sequencing validation (oRiboT-Tt2-ed) (Supplementary Fig. 3) was transformed with a plasmid encoding Cas9 from *S. pyogenes* under a pLtetO inducible promoter. Cells were inoculated from single colonies, and grown to mid-logarithmic growth in a shaking incubator at 34 °C with induction of Cas9. To induce expression of the lambda-red recombination proteins (Exo, Beta and Gam), cell cultures were shifted to 42 °C for 15 min and then immediately cooled on ice. In a 4 °C environment, 1 mL of cells was centrifuged at 16,000 × *g* for 30 s. The supernatant was removed and the cells resuspended in Milli-Q water. The cells were spun down, the supernatant was removed, and the cells were washed a second time. After a final 30 s spin, the supernatant was removed and dsDNA with 400nt-homology 5′ and 3′ of the Cas9 cut-sites was introduced in DNase-free water to the cell pellet, along with a plasmid containing a CRISPR array with two spacers specific for the 3’ terminus of the *Tetrahymena* intron and tether of oRiboT, respectively. The ssODN-cell mixture was transferred to a pre-chilled 1 mm gap electroporation cuvette (Bio-Rad) and electroporated using the following parameters: 1.8 kV, 200 V and 25 mF. LB-min medium (3 mL) was immediately added to the electroporated cells. The cells were recovered from electroporation and grown at overnight with induction of both Cas9 and CRISPR plasmids.

### Filtered-MAGE (f-MAGE)

MAGE was carried out as previously described^51^. Liquid cultures were inoculated from single colonies, and grown to mid-logarithmic growth in a shaking incubator at 34 °C. To induce expression of the lambda-red recombination proteins (Exo, Beta and Gam), cell cultures were shifted to 42 °C for 15 min and then immediately cooled on ice. In a 4 °C environment, 1 mL of cells was centrifuged at 16,000 × *g* for 30 s. The supernatant was removed and the cells were resuspended in Milli-Q water. The cells were spun down, the supernatant was removed, and the cells were washed a second time. After a final 30 s spin, the supernatant was removed and ssODNs prepared at a concentration of 5–6 μM in DNase-free water were added to the cell pellet. The ssODN-cell mixture was transferred to a pre-chilled 1 mm gap electroporation cuvette (Bio-Rad) and electroporated using the following parameters: 1.8 kV, 200 V and 25 mF. LB-min medium (3 mL) was immediately added to the electroporated cells. The cells were recovered from electroporation and grown at 30 °C for 3–3.5 h. Once cells reached mid-logarithmic growth they were used in additional MAGE cycles.

The ssODNs used for Filtered-MAGE were designed to possess between 0 to 70 nt of homology to the intron in order to determine optimum design parameters for targeted mutagenesis and reduction of off-target mutations. All mutagenesis was performed on a genomically integrated oRiboT-Tt1 integrated with clonetegration into C321.ΔA_spec (containing a 22-nt mutation 108-nt upstream of the intron-exon junction to distinguish it from native ribosomes) (Supplementary Fig. 4). For subsequent f-MAGE experiments, 44 nt overlap to intron was used, following the general MAGE protocol as above. For MAGE mutagenesis of RiboT plasmid for cell survival in antibiotics, the same oligo design strategy was employed and oligo homology was targeted to the 5′ or 3′ side of intron, respectively (Supplementary Fig. 5). Cells were recovered after every MAGE cycle. A minimum of 3 MAGE cycles were used due to lower efficiency of plasmid targeting. Cells were then recovered overnight before plating or selections.

### RNA isolation and RT-PCR

Cells expressing WT oRiboT or variants were grown overnight in LB media supplemented with 50 mg/mL carbenicillin, diluted 1:100, and grown to mid-logarithmic growth with aTc induction for RNA isolation. Total cellular RNA was purified using a DNeasy Blood and Tissue Kit (Qiagen) following the manufacturer’s instructions. RT-PCR was performed using SuperScript OneStep RT-PCR System with Platinum Taq DNA Polymerase on purified RNA from oRiboT, oRiboT-Tt1, oRiboT-Tt1∆, oRiboT-Tt4, oRiboT-Tt4∆. As a control and in order to rule out PCR amplification of the DNA template, RNA from cells containing oRiboT-Tt4 and oRiboT-Tt4∆ were treated with or without DNase. We hypothesize that if DNA template were present, the amplified PCR product would match the non-spliced intron instead of the size corresponding to the post-spliced RNA or WT ribosomes. As shown in Supplementary Fig. 18, samples containing DNase show the expected products for oRiboT-Tt4 and oRiboT-Tt4∆ whereas the same samples lacking DNase show an unspliced band suggestive of genomic DNA contamination. All other RT-PCR reactions were performed in the presence of DNase to eliminate genomic DNA contamination. Products of RT-PCR were analyzed by agarose gel electrophoresis and sequenced by Sanger sequencing.

*Testing oRiboT activity* in vivo C321.ΔA_spec cells were transformed with the following plasmids: (1) oRiboT and poGFP (2) oRiboT-Tt1 and poGFP (3) oRiboT-Tt1∆ and poGFP (4) oRiboT-Tt2 and poGFP (5) oRiboT-Tt2∆ and poGFP (6) oRiboT-Tt3 and poGFP (7) oRiboT-Tt3∆ and poGFP. All transformed strains were grown at 34 °C in LB-min medium supplemented with 50 mg/mL carbenicillin and 30 mg/mL of kanamycin. Wells of a 96-well plate were filled with 150 µL of LB media supplemented with 50 mg/mL carbenicillin and 30 mg/mL kanamycin. The wells were inoculated with colonies from each plasmid combination above (in triplicate) and incubated at 34 °C for 16 h with shaking. Clear bottom wells of another 96-well plate were filled with 150 µL of LB-min medium supplemented with 50 mg/mL carbenicillin and 30 mg/mL of kanamycin, and 1 mM IPTG and 100 ng/mL anhydrotetracycline (aTc). The plate was inoculated with 2 µL of saturated initial inoculation plate, and incubated with linear shaking (731 cycles per min) for 16 h at 34 °C on a Biotek Synergy H1 plate reader, with continuous monitoring of cell density (OD_600_) and sfGFP fluorescence (excitation at 485 nm and emission 528 nm with sensitivity setting at 70).

### Oligonucleotides and DNA sequencing

All oligonucleotides were purchased from Integrated DNA Technologies or the Yale University W.M. Keck Oligonucleotide Synthesis Facility with standard purification. MAGE oligonucleotides were 90 nucleotides in length and contained two phosphorothioated bases on the 5′ end^52^. Depending on the oligonucleotides described in the paper, degenerate bases or mutations were placed within the oligo (Supplementary Table 2). Additional primers were purchased for cloning and RT-PCR on oRiboT constructs. Primers for NGS were designed with five degenerate bases at the 5′ end. To create libraries for NGS, genomic DNA of each of ~2 × 10^9^ cells after f-MAGE was extracted using a Qiagen Genomic DNA purification kit and PCR was used for targeted amplification of the sequencing region. Up to two libraries were pooled for sequencing using an Illumina MiSeq. Data was analyzed with open source software^53^; briefly, after quality filtering, reads were searched for primer sequence, the site of mutagenesis was determined, and WT and mutant reads were quantified.

### Antibiotic selections

Following the f-MAGE procedure as described above, cells containing RiboT-intron or WT ribosome-intron plasmids were diversified with six cycles of f-MAGE, with degenerate ssODNs targeting regions 2030–2034 and 2057–2061, and having homology to the 5′ or 3′ portion of the intron, respectively (Supplementary Fig. 5, Supplementary Table 2). Cells were grown for 16 h with aTc to induce ribosome expression. Then 50 µL of each culture was seeded into 3 mL of LB-min medium containing aTc and antibiotic (273 μM erythromycin, 1.3 mM clindamycin, 7.74 μM chloramphenicol, or 28.22 mM lincomycin) and grown overnight. Plasmid DNA was isolated from each culture (Qiagen) and re-transformed into unselected C321 and MG1655 strains to confirm that the plasmid was causal to antibiotic resistance. The cells were plated on carbenicillin plates. Individual clones were grown in triplicates in a 96-well plate after overnight induction in aTc in LB, with aTc and one of four antibiotics included at the concentrations specified above.

### Anti-Shine-Dalgarno site editing experiments

Cells containing oRiboT-CTt2-Tt4 plasmid were diversified with six cycles of f-MAGE, with ssODN (Anti-SD-WT) that switches the orthogonal aSD to the WT *E. coli* aSD sequence targeting region in the 16S rRNA (Supplementary Fig. 11, Supplementary Table 2). Cells recovered from each of the cycles of f-MAGE were grown for 16 h with aTc and IPTG to induce ribosome and oGFP expression, respectively. The oGFP fluorescence of all members of the population was quantified with the BD FACSAria, using BD FACSDiva v.8.0 software for acquisition. FlowJo software was used for analysis. A gate was drawn in order to analyze cells of similar size and morphology. An example of the gating strategy used is provided in Supplementary Fig. 20.

### Multisite editing and evolution experiments

For multisite-editing experiments, cells containing oRiboT-CTt2-Tt4 plasmid were diversified with six cycles of f-MAGE with ssODNs to make the M4 mutation (Table 1) in the 23S rRNA, and having homology to the 5′ or 3′ portion of the CTt intron, respectively, as well as an ssODN to switch the orthogonal aSD to the WT *E. coli* aSD sequence targeting region in the 16S rRNA (Fig. 6a, Supplementary Table 2).

For multisite-evolution experiments, cells containing oRiboT-CTt2-Tt4 plasmid were diversified with six cycles of f-MAGE with ssODNs targeting regions 2030–2034 and 2057–2061 in the 23S rRNA, and having homology to the 5′ or 3′ portion of the CTt intron, respectively, as well as an ssODN to switch the orthogonal aSD to the WT *E. coli* aSD sequence targeting region in the 16S rRNA (Fig. 6a, Supplementary Table 2).

Cells recovered from each of the cycles of f-MAGE were grown for 16 h with aTc and IPTG to induce ribosome and oGFP expression, respectively. The oGFP fluorescence of cells across the population was quantified with the BD FACSAria, using BD FACSDiva v.8.0 software for acquisition. The FlowJo package was used for flow cytometry data analysis. For control experiments to dissect unique phenotypes via continues f-MAGE editing (Supplementary Fig. 19) cells from f-MAGE cycle 0-6 were FACS-sorted into negative and positive bins, respectively, and grown overnight. Confluent cultures were grown for 16 h with 100 ng/mL aTc to induce ribosome expression, and plated on chloramphenicol plates for obtaining CFU counts (see below).

For antibiotic selection experiments, cells from each of cycles of f-MAGE were grown for 16 h with 100 ng/mL aTc to induce ribosome expression. Fifty microliters of confluent culture was plated on LB-min agar plates containing 100 ng/mL aTc and 15.48 μM chloramphenicol, or LB-min agar plates containing 50 mg/mL carbenicillin and 30 mg/mL of kanamycin (non-selective plates). CFUs were quantified for the selective and non-selective plates to calculate survival ratios. In order to obtain kinetic growth curves, 1.5 µL of each overnight culture was seeded into 150 µL of LB-min medium containing 100 ng/mL aTc and 7.74 μM chloramphenicol in a 96-well clear bottom plate. The plate was incubated with linear shaking (731 cycles per min) for 16 h at 34°C on a Biotek Synergy H1 plate reader, with continuous monitoring of cell density (OD_600_).

### Reporting summary

Further information on research design is available in the Nature Research Reporting Summary linked to this article.

## Supplementary information

Supplementary Information

Reporting Summary

## Data Availability

All data generated or analyzed during this study are included in this published article (and supplementary information). The original raw NGS data from f-MAGE and f-CRISPR quantification experiments has been deposited at the Genome Sequence Archive (GSA) ID CRA005526 and can be found at: https://figshare.com/projects/Filtered_Editing/126737. Source data are provided with this paper.

## Source data

Source Data
