# Supplementary material for: Targeted editing and evolution of engineered ribosomes in vivo by filtered editing
Source: Nat Commun. 2022 Jan 10;13:180. doi: 10.1038/s41467-021-27836-x (PMC8748908; doi:10.1038/s41467-021-27836-x)
Supplement: Supplementary file 1 — Supplementary Information [file 41467_2021_27836_MOESM1_ESM.pdf]

Supplementary Information

Targeted editing and evolution of engineered ribosomes *in vivo* by filtered editing

Felix Radford[1,2], Shane D. Elliott[1], Alanna Schepartz[3,4], Farren J. Isaacs[1,2,5]

[1]Department of Molecular, Cellular, and Developmental Biology, Yale University, New Haven, CT 06520, USA
[2]Systems Biology Institute, Yale University, West Haven, CT 06516, USA
[3]Department of Chemistry, University of California, Berkeley, CA 94720, USA
[4]Department of Molecular and Cell Biology, University of California, Berkeley, CA 94720, USA
[5]Department of Biomedical Engineering, Yale University, New Haven, CT 06520, USA

*Correspondence should be addressed to farren.isaacs@yale.edu

Contents:

I. Supplementary Notes……………………………………………….2

Supplementary Note 1: Evolution of ribosomes with a panel of new antibiotic resistance mutations……………………………………2

Supplementary Note 2: Characterization of oRiboT with *Tetrahymena* intron introduced across sites 2, 3, and 4…………………………2

Supplementary Note 3: Engineering of Chimeric introns………..…3

II. Supplementary figures and tables…………………………………..…5

Supplementary table 1…………………………………… 5
Supplementary table 2…………………………………… 9
Supplementary table 3…………………………………… 10
Supplementary table 4…………………………………… 11
Supplementary table 5…………………………………… 12
Supplementary table 6…………………………………… 13
Supplementary Fig. 1…………………………………… 14

Supplementary Fig. 2.. 15
Supplementary Fig. 3.. 16
Supplementary Fig. 4.. 17
Supplementary Fig. 5.. 18
Supplementary Fig. 6.. 19
Supplementary Fig. 7.. 20
Supplementary Fig. 8.. 21
Supplementary Fig. 9.. 22
Supplementary Fig. 10..23
Supplementary Fig. 11..24
Supplementary Fig. 12..25
Supplementary Fig. 13..26
Supplementary Fig. 14..27
Supplementary Fig. 15..28
Supplementary Fig. 16..29
Supplementary Fig. 17..30
Supplementary Fig. 18..31
Supplementary Fig. 19..32
Supplementary Fig. 20..33
Supplementary References.....................................34

I. Supplementary Notes

Supplementary Note 1. Evolution of ribosomes with a panel of new antibiotic resistance mutations

We first used f-MAGE to recreate G2032A, G2057A, and A2058G mutations in non-orthogonal wild type (WT-Tt2) and tethered (RiboT-Tt2) ribosomes containing the *Tetrahymena* intron at Site 2. These mutations were chosen because they confer resistance to erythromycin, clindamycin, chloramphenicol, and lincomycin[1, 2]. We expressed the rRNA from the strong inducible PL-tetO promoter on plasmids to enhance ribosome expression. F-MAGE was used to introduce targeted modifications by directing mutagenic ssODNs at the 5' and 3' ribosome-intron junctions at Site 2 (**Supplementary Fig. 5b**). WT-Tt2-derived mutants at the three published sites displayed the expected antibiotic resistance phenotypes when challenged with the panel of four antibiotics **(Supplementary Figs. 6-9, top panels)**. These results demonstrate cell survival from WT-Tt2 ribosomes under antibiotic conditions that render the native ribosomes non-functional, and validate two key aspects of our study. First, new antibiotic-resistant ribosomes could be evolved by f-MAGE in the presence of the native translational machinery. Second, cells are capable of surviving solely from ribosomes transcribed from intron-containing genes. Interestingly, we observed differences in antibiotic sensitivities in RiboT-Tt2 (*e.g.,* resistance to erythromycin in native RiboT-Tt2 variant), suggesting that the presence of the tether may cause functional changes in the ribosome that are not completely understood. (**Supplementary Figs. 6-9, top panels**).

We next applied f-MAGE to generate a complex library of RiboT-Tt2 ribosomes in order to discover new mutations that confer antibiotic resistance. Based on the positions of known antibiotic resistance mutations, we designed mutagenic MAGE ssODNs containing five degenerate nucleotides to target two 23S rRNA regions: Region 1: 2030 - 2034 and Region 2: 2057 - 2061 **(Supplementary Fig. 5b)**. We performed six cycles of MAGE with this complex pool of ssODNs, followed by liquid selections in the four antibiotics to isolate individual mutants after plating on solid media **(Supplementary Fig. 5a)**. We identified seven ribosomal mutants with varying degrees of resistance to the antibiotics **(Table 1).** To confirm that these ribosome mutants conferred antibiotic resistance and that our results were independent of any potential mutation(s) in the cell's native ribosome or elsewhere, we re-transformed the plasmids containing the mutant ribosomes into a clean MG1655 genetic background and found that each mutant conferred resistance to a subset of the antibiotics (**Supplementary Figs. 6-10)**. For example, some of the identified mutants exhibited broad resistance to the panel of four antibiotics assayed (*e.g.,* M4, M6), while others (*e.g.,* M5, M7) showed resistance exclusive to one or two antibiotics.

To further validate these RiboT antibiotic-resistant mutants and assess the impact of the tethered linker, we reconstructed these mutations in the natural ribosome with the intron (WT-Tt2) (Covered in main text, section: "Application of filtered editing for evolution of new ribosomes resistant to antibiotics").

Supplementary Note 2. Characterization of oRiboT with *Tetrahymena* intron introduced across sites 2, 3, and 4

Having demonstrated that oRiboT maintains its function upon introduction of the *Tetrahymena* intron into site 1, and that the *Tetrahymena* intron is completely spliced out and the rRNA is scarlessly ligated, we next sought to determine whether the *Tetrahymena* intron could

be placed in multiple distinct positions in the ribosome. We constructed three additional oRiboT constructs in which the intron was inserted at two additional sites within the large subunit (sites 2 and 3) (**Supplementary Table 1, Fig. 1b**), or in the small subunit (site 4). We profiled these variants (Site 2 = oRiboT-Tt2, Site 3 = oRiboT-Tt3, site 4 = oRiboT-Tt4) with respect to oGFP expression under the same growth and induction conditions described for oRiboT-Tt1 and observed qualitatively equivalent results **(Supplementary Fig. 14)**. Importantly, oRiboT-Tt2, oRiboT-Tt3, and oRiboT-Tt4 all supported robust expression of oGFP, indicating that the introns were successfully spliced to yield functional ribosomes (**Fig. 2e**). We also performed RT-PCR on total purified RNA from strains with a *Tetrahymena* intron at sites 1 - 4, as above, and observed a single band indicating complete splicing at each site **(Supplementary Fig. 15)**. Sequencing of the RT-PCR products confirmed the scarless ligation of the ribosome at the intron-exon junction post-splicing in all four insertion sites.

To further characterize the *Tetrahymena* ribosomes, we used oRiboT-Tt1, oRiboT-Tt2, and oRiboT-Tt3 as templates to construct ΔIGS mutants of each, yielding oRiboT-Tt1Δ, oRiboT-Tt2Δ, and oRiboT-Tt3Δ, respectively. As expected, all ΔIGS oRiboT mutants showed low levels of GFP fluorescence, demonstrating the deleterious impact on translation of an unspliced *Tetrahymena* intron (**Supplementary Fig. 14**) in contrast to full ribosomal function upon splicing of the un-ablated *Tetrahymena* intron at sites throughout the large or small subunits of the ribosome. These results indicated that we could modularly place introns into the ribosome across many sites, opening its use for filtered editing at significant ribosomal positions.

Supplementary Note 3. Engineering of Chimeric introns

In order to create orthogonal introns with both the capability to splice in any location (as demonstrated for the *Tetrahymena* intron) and also contain unique 5' or 3' sequences that would differentiate these introns from each other, we created chimeric introns by toggling between elements from one intron to another **(Fig. 5a)**. Changes to P1 have been shown to be extremely deleterious to *Tetrahymena* function[3] but we hypothesized that P1 transplanted from introns demonstrating self-splicing activity could functionally compensate for the loss of the native P1 in *Tetrahymena*. We created an oRiboT-variant with chimeric *Tetrahymena* introduced into site 2, containing P1 from Tfa (oRiboT-CTt2) **(Fig. 5a)**. We constructed strains with an oGFP reporter controlled by the IPTG—LacR inducible PL-lacO promoter and oRiboT from the aTc—TetR inducible PL-tetO promoter, containing WT oRiboT, oRiboT-Tt2, or oRiboT-CTt2. Upon assaying steady-state GFP expression under full induction (+IPTG +aTc), we found that the ribosome produced oGFP at the same level as WT oRiboT or oRiboT-Tt2 **(Fig. 5b)**. This result suggests that not only can chimeric introns combining elements of *Tetrahymena* with other group I introns be functional, but also that oRiboT containing the engineered CTt intron has WT-level activity.

Encouraged by this result, we constructed three additional chimeric introns based on CTt **(Supplementary Fig. 15)**, in which in addition to the chimeric P1, we varied the P9 helix so as to create a unique 3' homology region. We constructed two variants (CTt9a and CTt9b) where the P9.1- P9.2 helix from Tfa or Tfb, respectively, replaced the *Tetrahymena* P9.2 in CTt **(Fig. 5a)**. In addition, we built a chimeric intron (CTt9c) where we altered the native sequence of bases in the *Tetrahymena* P9.2 in CTt while maintaining homology across the stem loop, so as to maintain the correct secondary structure but create a unique sequence. It has been known for over 20 years that P9.1 – P9.2 are not essential for *Tetrahymena* intron catalytic activity, and in

fact can be deleted completely without compromising intron function[4]. However, upon assaying oGFP activity in oRiboT-CTt9a, oRiboT-CTt9b, or oRiboT-CTt9c, we found that the ribosome was not functional **(Fig. 5b)**. Furthermore, deletion of P9.1-P9.2 from oRiboT-Tt-2 **(Supplementary Fig. 17a)** also led to a non-functional ribosome **(Supplementary Fig. 17b)**. These data suggest that not only is the catalytic efficiency of Tt important, but the respective relative rates of intron and ribosome folding are crucial for the maturation of functional ribosomes; indeed deletion of P9.1-P9.2 was shown to decrease *Tetrahymena* intron folding kinetics[5]. Helices P9.1 –P9.2 have a role in stabilizing folding of the catalytic core, and replacing them with another structure could destabilize this process. Nevertheless, having chimeric introns with P1 helix would allow for a modular strategy to target multiple parallel sites in the ribosome or other noncoding RNAs. We thus proceeded with further characterization of the CTt intron.

We performed RT-PCR on total purified RNA from *E. coli* strains containing wild type (WT) oRiboT (+ control), oRiboT-Tt2 (+ control), or oRiboT-CTt2 using primers that amplified the region spanning the intron-exon junction. A single band was observed in all cases **(Fig. 5c)**, suggesting the complete and scarless splicing of the engineered intron from oRiboT-CTt2, just as the natural intron was spliced from oRiboT-Tt2. Sequencing of oRiboT-CTt2 RT-PCR products confirmed the scarless ligation of the ribosome at the intron-exon junction post-splicing. Furthermore, this indicates that the ribosome assembled correctly, and is functional after intron splicing.

Having unique introns (*i.e.* Tt and CTt) that could be tolerated in multiple positions in the ribosome, we next sought to employ filtered editing for continuous *in vivo* multi-site evolution. We chose to position an intron at site 2, so we could diversify the PTC/exit tunnel, and also an intron near the anti-Shine-Dalgarno Sequence (aSD) in the 16S rRNA (which we designate site 4) **(Fig. 1b)** in order to evolve orthogonality of the aSD simultaneously *in vivo* **(Supplementary Fig. 11)**. As before, we expressed the rRNA from the strong inducible PL-tetO promoter on plasmids to enhance ribosome expression, in a strain containing an oGFP reporter under the IPTG—LacR inducible PL-lacO promoter. To first validate whether a ribosome containing such a placement of introns would be functional, we inserted the chimeric CTt intron into site 2 of oRiboT, and natural Tt intron into site 4 to form oRiboT-CTt2-Tt4. Upon full induction (+IPTG +aTc), we confirmed that oRiboT-CTt2-Tt4 also produced oGFP at the same level as WT oRiboT. These results suggested efficient intron self-splicing and ribosome assembly **(Fig. 5d)**. To conclusively validate complete splicing of both introns within oRiboT-CTt2-Tt4, we performed the RT-PCR assay as before on total purified RNA from *E. coli* strains containing wild type (WT) oRiboT(+ control), oRiboT-Tt2 (+ control), or oRiboT-CTt2-Tt4 using primers that amplified the region spanning the intron-exon junctions of each intron. Analysis, and subsequent sequencing, of the RT-PCR products indicated complete splicing at both sites 2 and 4, and seamless ligation of exon. A single band (~128nt) was observed for the site 2 and 4 introns, respectively, with no evidence of the unspliced (535 nt) product **(Fig. 5e)**.

II. Supplementary figures and tables

Construct	Ancestor or Source	Notes	Use
pRibo-T	Kindly provided by M. Jewett[6]	Used to create RiboT constructs in this study	
poRibo-T	Kindly provided by M. Jewett[7]	Used to create oRiboT constructs in this study	
pAM522	Kindly provided by M. Jewett[7]	Used to create untethered ribosome constructs in this study	antibiotic experiments
poGFP	Kindly provided by M. Jewett[7]	sfGFP under PL-lacO inducible promoter, and containing an orthogonal ribosome binding site.	*in vivo* translation assays
pLtetO-RiboT	pRibo-T	PL-tetO inducible RiboT,with ColE1 origin of replication	antibiotic experiments
pLtetO-oRiboT	poRibo-T	PL-tetO inducible oRiboT, with ColE1 origin of replication	splicing investigation, *in vivo* translation assays
oRiboT-Tt1	pLtetO-oRiboT	pLtetO-oRiboT with *Tetrahymena* intron placed into site 1	splicing investigation, *in vivo* translation assays
oRiboT-Tt1Δ	oRiboT-Tt1	oRiboT-Tt1 with IGS deletion	splicing investigation, *in vivo* translation assays
oRiboT-Tt2	pLtetO-oRiboT	pLtetO-oRiboT with *Tetrahymena* intron placed into site 2	splicing investigation, *in vivo* translation assays
oRiboT-Tt2-ed	oRiboT-Tt2	oRiboT-Tt2 genomically integrated with distinguishing mutation for NGS analysis	f-CRISPR experiments
RiboT-Tt2	oRiboT-Tt2		antibiotic experiments
oRiboT-Tt2Δ	oRiboT-Tt2	oRiboT-Tt2 with IGS deletion	splicing investigation, *in vivo* translation assays
oRiboT-Tt3	pLtetO-oRiboT	pLtetO-oRiboT with *Tetrahymena* intron placed into site 3	splicing investigation, *in vivo* translation assays

oRiboT-Tt3Δ	oRiboT-Tt3	oRiboT-Tt3 with IGS deletion	splicing investigation, *in vivo* translation assays
oRiboT-Tt4	pLtetO-oRiboT	pLtetO-oRiboT with *Tetrahymena* intron placed into site 4	*in vivo* translation assays
oRiboT-Tt1b	oRiboT-Tt1	oRiboT-Tt1 with distinguishing mutation introduced for sequencing	splicing investigation
oRiboT-Tt1bΔ	oRiboT-Tt1Δ	oRiboT-Tt4 with IGS deletion	splicing investigation
oRiboT-Np1	pLtetO-oRiboT	pLtetO-oRiboT with *Nostoc punctiforme* intron placed into site 1	multi-site Filtered Editing
oRiboT-Ba1	pLtetO-oRiboT	pLtetO-oRiboT with *Bacillus anthracis* intron placed into site 1	multi-site Filtered Editing
oRiboT-Tfa1	pLtetO-oRiboT	pLtetO-oRiboT with *Tilletiopsis flava-515* intron placed into site 1	multi-site Filtered Editing
oRiboT-Tfb1	pLtetO-oRiboT	pLtetO-oRiboT with *Tilletiopsis flava-1199* intron placed into site 1	multi-site Filtered Editing
oRiboT-Tfc1	pLtetO-oRiboT	pLtetO-oRiboT with *Tilletiopsis flava-1506* intron placed into site 1	multi-site Filtered Editing
oRiboT-T71	pLtetO-oRiboT	pLtetO-oRiboT with T7-like bacteriophage Phi DNA polymerase intron intron placed into site 1	multi-site Filtered Editing
oRiboT-Az1	pLtetO-oRiboT	pLtetO-oRiboT with *Azoarcus* intron placed into site 1	multi-site Filtered Editing
oRiboT-Pc1	pLtetO-oRiboT	pLtetO-oRiboT with *Pneumocystis carinii* intron placed into site 1	multi-site Filtered Editing
oRiboT-Ag1	pLtetO-oRiboT	pLtetO-oRiboT with *Agrobacterium* intron placed into site 1	multi-site Filtered Editing

oRiboT-T41	pLtetO-oRiboT	pLtetO-oRiboT with Bacteriophage T4 sunY intron placed into site 1	multi-site Filtered Editing
oRiboT-Np2	pLtetO-oRiboT		multi-site Filtered Editing
oRiboT-Ba2	pLtetO-oRiboT		multi-site Filtered Editing
oRiboT-Tfa2	pLtetO-oRiboT		multi-site Filtered Editing
oRiboT-Tfb2	pLtetO-oRiboT		multi-site Filtered Editing
oRiboT-Tfc2	pLtetO-oRiboT		multi-site Filtered Editing
oRiboT-T72	pLtetO-oRiboT		multi-site Filtered Editing
oRiboT-Az2	pLtetO-oRiboT		multi-site Filtered Editing
oRiboT-Pc2	pLtetO-oRiboT		multi-site Filtered Editing
oRiboT-Ag2	pLtetO-oRiboT		multi-site Filtered Editing
oRiboT-T42	pLtetO-oRiboT		multi-site Filtered Editing
oRiboT-T41-Tt2	oRiboT-T41	pLtetO-oRiboT with intron T4 at site 1 and intron Tt2 at site 2	multi-site Filtered Editing
oRiboT-Ag1-Tt2	oRiboT-Ag1	pLtetO-oRiboT with intron Ag at site 1 and intron Tt2 at site 2	multi-site Filtered Editing
oRiboT-Pc1-Tt2	oRiboT-Pc1	pLtetO-oRiboT with intron Pc at site 1 and intron Tt2 at site 2	multi-site Filtered Editing
oRiboT-Az1-Tt2	oRiboT-Az1	pLtetO-oRiboT with intron Az at site 1 and intron Tt2 at site 2	multi-site Filtered Editing
oRiboT-T7-Tt2	oRiboT-T71	pLtetO-oRiboT with intron T7 at site 1 and intron Tt2 at site 2	multi-site Filtered Editing
oRiboT-Tfa1-Tt2	oRiboT-Tfa1	pLtetO-oRiboT with intron Tfa at site 1 and intron Tt2 at site 2	multi-site Filtered Editing
oRiboT-Tfb1-Tt2	oRiboT-Tfb1	pLtetO-oRiboT with intron Tfb at site 1 and intron Tt2 at site 2	multi-site Filtered Editing
oRiboT-Tfc1-Tt2	oRiboT-Tfc1	pLtetO-oRiboT with intron Tfc at site 1 and intron Tt2 at site 2	multi-site Filtered Editing
oRiboT-T41-Tt3	oRiboT-T41		multi-site Filtered Editing
oRiboT-Ag1-Tt3	oRiboT-Ag1		multi-site Filtered Editing
oRiboT-Pc1-Tt3	oRiboT-Pc1		multi-site Filtered Editing
oRiboT-Az1-Tt3	oRiboT-Az1		multi-site Filtered Editing
oRiboT-T7-Tt3	oRiboT-T71		multi-site Filtered Editing
oRiboT-Tfa1-Tt3	oRiboT-Tfa1		multi-site Filtered Editing

oRiboT-Tfb1-Tt3	oRiboT-Tfb1		multi-site Filtered Editing
oRiboT-Tfc1-Tt3	oRiboT-Tfc1		multi-site Filtered Editing
oRiboT-T41-Tt4	oRiboT-T41		multi-site Filtered Editing
oRiboT-Ag1-Tt4	oRiboT-Ag1		multi-site Filtered Editing
oRiboT-Pc1-Tt4	oRiboT-Pc1		multi-site Filtered Editing
oRiboT-Az1-Tt4	oRiboT-Az1		multi-site Filtered Editing
oRiboT-T7-Tt4	oRiboT-T71		multi-site Filtered Editing
oRiboT-Tfa1-Tt4	oRiboT-Tfa1		multi-site Filtered Editing
oRiboT-Tfb1-Tt4	oRiboT-Tfb1		multi-site Filtered Editing
oRiboT-Tfc1-Tt4	oRiboT-Tfc1		multi-site Filtered Editing
oRiboT-CTt2	pLtetO-oRiboT	pLtetO-oRiboT with chimeric intron (P1of *Tilletiopsis flava-515* intron merged *Tetrahymena* intron) placed into site 2	multi-site Filtered Editing
oRiboT-CTt9a	oRiboT-CTt2	pLtetO-oRiboT with chimeric intron CTt9a placed into site 2	
oRiboT-CTt9b	oRiboT-CTt2	pLtetO-oRiboT with chimeric intron CTt9b placed into site 2	
oRiboT-CTt9c	oRiboT-CTt2	pLtetO-oRiboT with chimeric intron CTt9c placed into site 2	
oRiboT-TtP9Δ	oRiboT-Tt2	pLtetO-oRiboT with intron TtP9Δ placed into site 2	
oRiboT-CTt3	pLtetO-oRiboT	pLtetO-oRiboT with intron CTt at site 3	multi-site Filtered Editing
oRiboT-CTt2-Tt4	oRiboT-CTt2	pLtetO-oRiboT with intron CTt at site 2 and intron Tt4 at site 4	multi-site Filtered Editing
oRiboT-CTt3-Tt4	oRiboT-CTt3	pLtetO-oRiboT with intron CTt at site 3 and intron Tt4 at site 4	multi-site Filtered Editing

Supplementary Table 1. Constructs used in this study.

Name	Description	ssODN Sequence (5'-3')
Determination of maximum design parameters for f-MAGE ssODN design		
I70R20	70nt overlap to intron and 20nt overlap to 23S RNA	a*c*aaggaaCttcgctaccttCGAGTACTCCAAAACTAATCAATATACTTTCGCATACAAATTAGTTCCCAGCGGCTCCAGTGTTGCATCA
I60R30	60nt overlap to intron and 30nt overlap to 23S RNA	a*a*cttacccgacaaggaaCttcgctaccttCGAGTACTCCAAAACTAATCAATATACTTTCGCATACAAATTAGTTCCCAGC
I50R40	50nt overlap to intron and 40nt overlap to 23S RNA	t*g*caggtcggaacttacccgacaaggaaCttcgctaccttCGAGTACTCCAAAACTAATCAATATACTTTCGCATACAAATTAGTTCCCA
I44R46	44nt overlap to intron and 46nt overlap to 23S RNA	c*a*ttcgtgcaggtcggaacttacccgacaaggaaCttcgctaccttCGAGTACTCCAAAACTAATCAATATACTTTCGCATACAAATTAG
I35R56	35nt overlap to intron and 56nt overlap to 23S RNA	t*c*attacgccattcgtgcaggtcggaacttacccgacaaggaaCttcgctaccttCGAGTACTCCAAAACTAATCAATATACTTTCGCAT
I30R70	30nt overlap to intron and 70nt overlap to 23S RNA	g*g*ccatcattacgccattcgtgcaggtcggaacttacccgacaaggaaCttcgctaccttCGAGTACTCCAAAACTAATCAATATACTTT
I20R70	20nt overlap to intron and 70nt overlap to 23S RNA	g*a*gacagcctggccatcattacgccattcgtgcaggtcggaacttacccgacaaggaaCttcgctaccttCGAGTACTCCAAAACTAATC
I15R75	15nt overlap to intron and 75nt overlap to 23S RNA	g*g*gtggagacagcctggccatcattacgccattcgtgcaggtcggaacttacccgacaaggaaCttcgctaccttCGAGTACTCCAAAAC
I10R80	10nt overlap to intron and 80nt overlap to 23S RNA	g*t*ctcgggtggagacagcctggccatcattacgccattcgtgcaggtcggaacttacccgacaaggaaCttcgctaccttCGAGTACTCC
R90.A	No overlap to intron	t*t*tcactgagtctcgggtggagacagcctggccatcattacgccattcgtgcaggtcggaacttacccgacaaggaaCttcgctacctt
R90.B	No overlap to intron	a*a*ggtagcgaaattccttgtcgggtaagttccgacctgcacgaaCggcgtaatgatggccaggctgtctccacccgagactcagtgaaat
Recapitulating known antibiotic mutations in pLtetO-RiboT and untethered ribosomes		
G2032A	Introduces G2032A mutation in 23S RNA	T*A*GCTACCAGGTGCATGCCTGATAACTTTTcagtgCAAAGGTAAATATTGCTATTTacactgcatTttcacagcgagttcaatttcact
G2057A	Introduces G2057A mutation in 23S RNA	A*a*gctatagtaaaggttcacggggtcttttcgtcttgccgcgggtCGAGTACTCCAAAACTAATCAATATACTTTCGCATACAAATTAGT
A2058G	Introduces A2058G mutation in 23S RA	t*c*agtgtcaagctatagtaaaggttcacggggtcttCccgtcttgccgcgggtCGAGTACTCCAAAACTAATCAATATACTTTCGCATAC
Creating library of mutants in sites 2030 - 2034 and 2057 – 2061 to evolve antibiotic resistance		
2030-2034	Diversify ribosome bases 2030 – 2034	C*T*GATAACTTTTcagtgCAAAGGTAAATATTGCTATTTacactgcNNNNNcacagcgagttcaatttcactgagtctcgggtggagacag
2057 – 2061	Diversify ribosome bases 2057 – 2061	C*a*agctatagtaaaggttcacggggtNNNNNcgtcttgccgcgggtCGAGTACTCCAAAACTAATCAATATACTTTCGCATACAAATTAG
In vivo f-MAGE on dual intron oRiboT		
AntiSD-WT	Changing anti-oRBS to WT anti-SD in oRiboT-sunY-*Tetrahymena*	t*g*tgagcactacaaagtacgcttctttaaggtaAGGAGGtgatccaaccgcCGAGTACTCCAAAACTAATCAATATACTTTCGCATACAA

Supplementary Table 2. ssDNA MAGE oligonucleotides used in this study. Single-stranded DNA Oligonucleotides were purchased from Integrated DNA Technologies (IDT) with two phosphorothioate bonds at the 5' end of ssODN (denoted by *). The degenerate base N represents all four bases.

Distinguish oRiboT RT-PCR product from WT rRNA					
Distinguishing sequence	Location in 23S sequence	WT sequence	Forward primer (5'-3')	Reverse primer (5'-3')	Found in ribosome variants:
tacgag	1929-1934	ggtagc	acggtgtgacgcctgcccgg	GTGATGCTATCAGACTGGTTCGCTGCTGCTCTCTAAAGTAcgacaaggaatttcctcgta	oRiboT-Tti1b oRiboT-Tti1bΔ
Selectively amplify genomic oRiboT-Tt1 or native ribosomes					
Distinguishing sequence	Location in 23S sequence	WT sequence	Forward primer (5'-3')	Reverse primer (5'-3')	Found in ribosome variants:
acagtcaattggcgtcgactac	1797-1818	gtgcaaacacgaaagtggacgt	gtgcaaacacgaaagtggacgt (WT)	ctcctgatgtccgaccaggatt	Genomically integrated oRiboT-Tt1
			agtcggacctctccttaatggga (oRiboT)		

Supplementary Table 3. Distinguishing sequences used for selectively amplifying oRiboT constructs in RT-PCR and f-MAGE optimization experiments.

RiboT construct	Ancestor or Source	Notes
RiboT-Tt2-2032A	RiboT-Tt2	G2032A mutation in RiboT-Tt2; made with f-MAGE
RiboT-Tt2-2057A	RiboT-Tt2	G2057A mutation in RiboT-Tt2; made with f-MAGE
RiboT-Tt2-2058G	RiboT-Tt2	A2058G mutation in RiboT-Tt2; made with f-MAGE
WT-Tt2	pAM522	
WT-Tt2-2032A	WT-Tt2	A2032A mutation in WT-Tt2; made with f-MAGE
WT-Tt2-2057A	WT-Tt2	A2057A mutation in WT-Tt2; made with f-MAGE
WT-Tt2-2058G	WT-Tt2	A2058G mutation in WT-Tt2; made with f-MAGE
RiboT-Tt2-M1	RiboT-Tt2	Isolated from antibiotic selections on library generated with f-MAGE
RiboT-Tt2-M2	RiboT-Tt2	Isolated from antibiotic selections on library generated with f-MAGE
RiboT-Tt2-M3	RiboT-Tt2	Isolated from antibiotic selections on library generated with f-MAGE
RiboT-Tt2-M4	RiboT-Tt2	Isolated from antibiotic selections on library generated with f-MAGE
RiboT-Tt2-M5	RiboT-Tt2	Isolated from antibiotic selections on library generated with f-MAGE
RiboT-Tt2-M6	RiboT-Tt2	Isolated from antibiotic selections on library generated with f-MAGE
RiboT-Tt2-M7	RiboT-Tt2	Isolated from antibiotic selections on library generated with f-MAGE
WT-Tt2-M1	WT-Tt2	Constructed with Gibson Assembly
WT-Tt2-M2	WT-Tt2	Constructed with Gibson Assembly
WT-Tt2-M3	WT-Tt2	Constructed with Gibson Assembly
WT-Tt2-M4	WT-Tt2	Constructed with Gibson Assembly
WT-Tt2-M5	WT-Tt2	Constructed with Gibson Assembly
WT-Tt2-M6	WT-Tt2	Constructed with Gibson Assembly
WT-Tt2-M7	WT-Tt2	Constructed with Gibson Assembly

Supplementary Table 4. Ribosome constructs generated with f-MAGE for antibiotic experiments, and new mutants isolated from selections in erythromycin, clindamycin, chloramphenicol, and lincomycin. Previously-known antibiotic-resistance mutations were recapitulated with f-MAGE in RiboT and untethered ribosome backgrounds, respectively. Seven new mutants were isolated from antibiotic selections on a diverse library generated in sites 2030 - 2034 and 2057 – 2061 in RiboT-Tti2, and those mutations were recapitulated in WT-Tt2 by traditional cloning.

Intron Variant	Abbreviation	Reference
Tetrahymena thermophila	Tt	[15]
Nostoc punctiforme	Np	[8]
Bacillus anthracis	Ba	[9]
Tilletiopsis flava-515	Tfa	[10]
Tilletiopsis flava-1199	Tfb	[10]
Tilletiopsis flava-1506	Tfc	[10]
T7-like bacteriophage Phi DNA polymerase intron	T7	[11]
Azoarcus	Az	[12]
Pneumocystis carinii	Pc	[13]
Agrobacterium	Ag	[14]
Bacteriophage T4 sunY intron	T4	[15]
Chimeric intron: P1Tfa into Tt	CTt	This study
Chimeric intron: P1Tfa into Tt and P9 from Tfa	CTt9a	This study
Chimeric intron: P1Tfa into Tt and P9 from Tfb	CTt9b	This study
Chimeric intron: P1Tfa into Tt and P9 mutant from Tt	CTt9c	This study

Supplementary Table 5. Introns employed in this study.

	Mutate genomic locus	Mutate episomal locus	*In vivo* mutagenesis	Generate randomized stretches of sequence	Make defined edits	Distinguish repetitive genetic elements	Minimal time between selection/screen of repetitive genetic element to next round of mutagenesis	Maximum *in vivo* complexity generated within locus per round of mutagenesis	References
**Filtered Editing**	**Y**	**Y**	**Y**	**Y**	**Y**	**Y**	**2 hrs**	**$^{**}10^9$**	This paper
CRISPR/Cas9	Y	Y	Y	Y	Y	N	NA	10^8	16-18 19
Base Editing	Y	NI	Y	N	Y	N	NA	NA	18
Prime Editing	Y	NI	Y	N	Y	N	NA	NA	20
MAGE	Y	Y	Y	Y	Y	N	NA	10^9	21
Overlap PCR	*N	Y	N	Y	Y	Y	2-5 days	10^6-10^7	22
Error-Prone PCR	N	Y	N	Y	N	Y	2-5 days	10^6-10^7	23
Quikchange Mutagenesis	N	Y	N	Y	Y	Y	2-5 days	10^6-10^7	24, 25
MP6 Random Mutagenesis	Y	Y	Y	Y	N	N	NA	10^1	26
evolvR	Y	NI	Y	Y	N	N	NA	**10^3	27
PACE	N	Y	Y	Y	N	N	NA	**10^2	28

Supplementary Table 6. Comparison of filtered editing with other techniques for performing editing and evolving repetitive genetic elements. For the challenge of editing and evolving repetitive genetic elements such as ribosomes to expand their functions, the previous toolkit available involved either a compromise of specificity, time, and/or depth of library complexity. Filtered editing for the first time enables the application of genome editing technologies to precisely edit and evolve repetitive genetic elements *in vivo*. Annotations in table: NI = not demonstrated at date of this publication. *Does not perform edits directly in genome, but can be used to generate dsDNA for genome engineering. ** Estimated values.

Targeting genome editing at repetitive genetic elements:

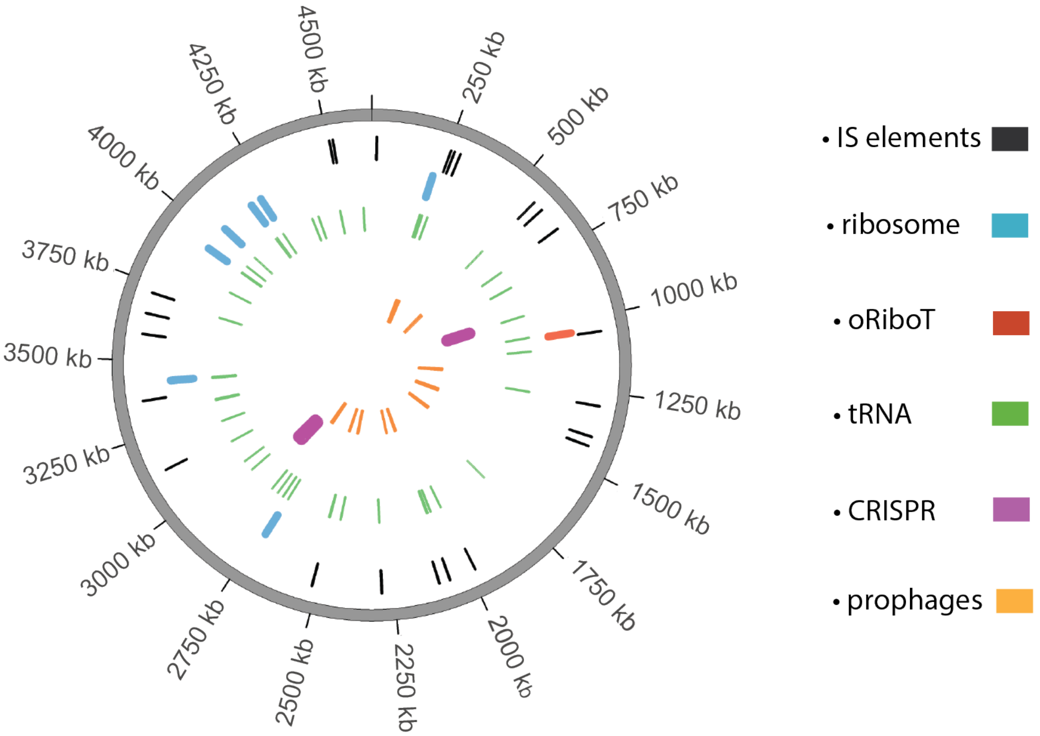

Supplementary Fig. 1. Examples of repetitive genetic elements present in the *E. coli* genome. The presence of repetitive elements in the genome presents a challenge to genome engineering, especially in the case of non-coding RNA. Repetitive sequences and extensive secondary structure of many noncoding RNAs may reduce MAGE efficiency and bias sequences toward favored secondary structures.

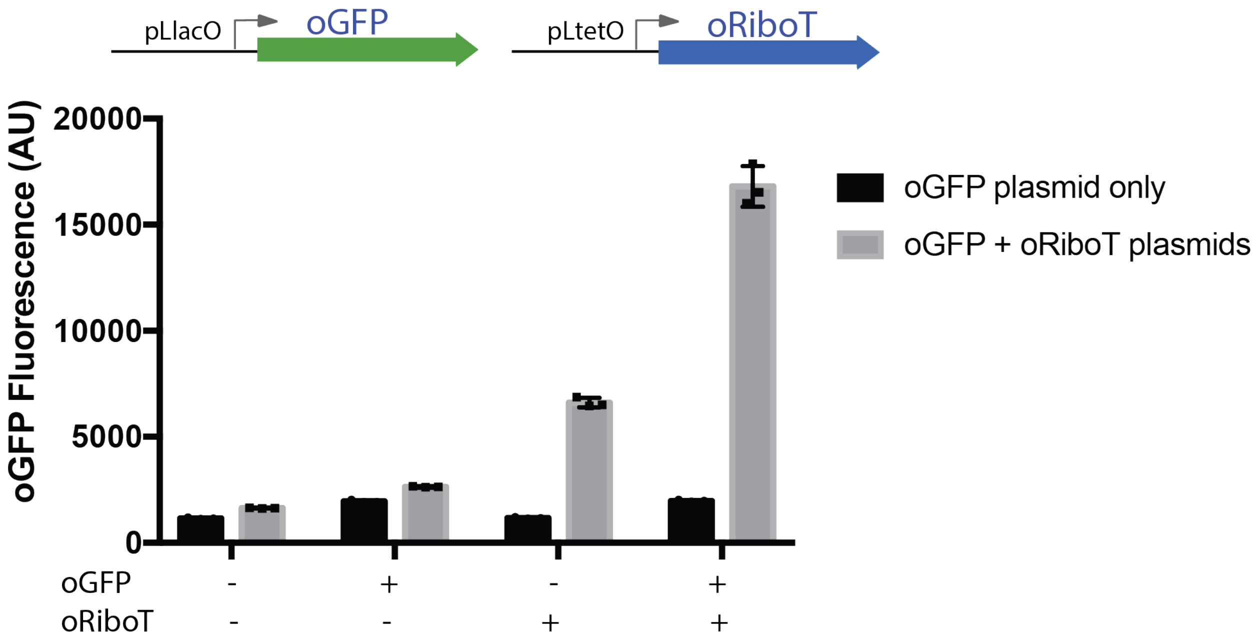

Supplementary Fig. 2. Comparison of oGFP expression by oRiboT and WT ribosomes. The level of oGFP fluorescence from cells containing poGFP only (black bars) or poGFP and pLtetO-RiboT plasmids (grey bars) with or without induction by IPTG (for oGFP) and aTc (for oRiboT). Values and error bars represent the mean and standard deviation of n=3 biologically independent replicates (dots). See Supplementary Table 2 for constructs.

a

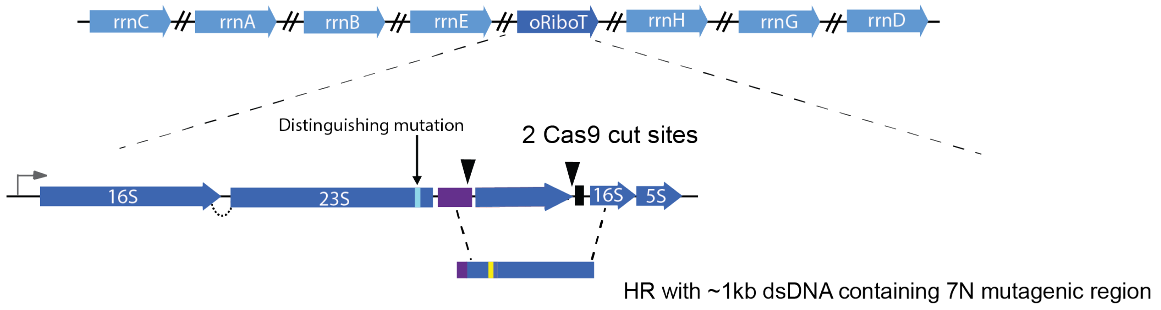

b

oRiboT			WT ribosomes		
WT	Mut	ARF	WT	Mut	ARF
752	42831	98.27%	42,010	124	0.29%

Supplementary Fig. 3. Calculated f-CRISPR efficiency. (a) A representation of the genomic ribosomal construct used for f-CRISPR editing of oRiboT-Tt2-ed. **(b)** Quantification of targeting of oRiboT versus WT ribosomes with f-CRISPR to introduce 7-N degenerate mutation downstream of site 2 (WT sequence: C2072-C2078).

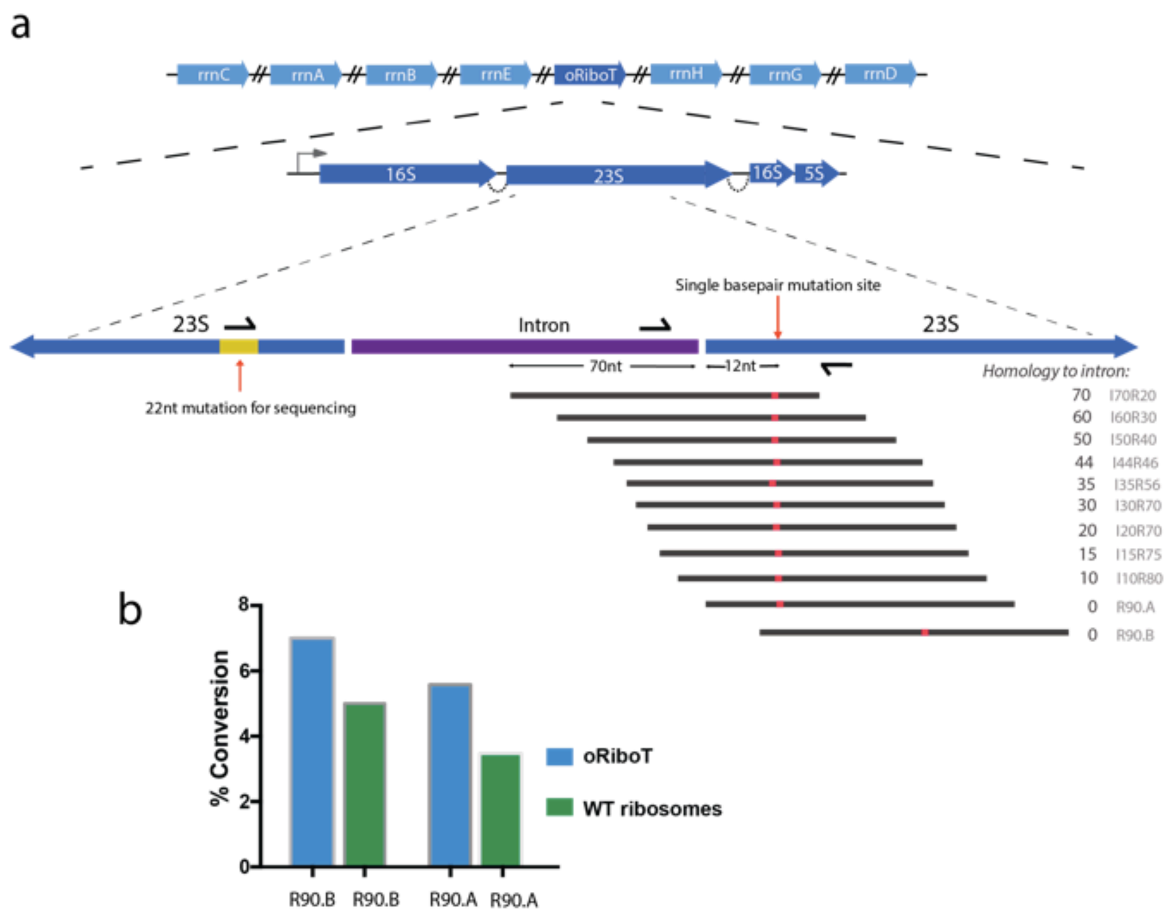

c

ssODN	oRibo-T			WT ribosomes		
	WT	Mut	ARF	WT	Mut	ARF
I70R20	53899	609	1.1%	45677	3	0.0066%
I60R30	50944	2830	5.3%	66124	104	0.2%
I50R40	47642	4256	8.2%	60073	279	0.44%
I44R46	48446	5966	11.0%	56547	678	1.18%
I35R56	55317	5575	9.2%	49988	818	1.61%
I30R70	60453	5869	8.9%	39841	675	1.67%
I20R70	42378	3493	7.6%	47477	1476	3.02%
I15R75	55906	5534	9.0%	49085	2090	4.08%
I10R80	54169	3938	6.8%	44949	1650	3.54%
R90.A	63702	3767	5.6%	50398	1811	3.50%

Supplementary Fig. 4. Determination of optimum homology between MAGE oligonucleotides and introns. (a) A representation of the genomic ribosomal construct and MAGE oligonucleotides used with homology arms of different lengths. Sequencing primers for WT ribosomes and oRiboT are indicated. **(b)** Determining the specificity of MAGE without the targeting intron. A MAGE oligonucleotide having equal targeting of WT ribosomes and oRiboT was used in order to quantify the level of targeting at native genomic ribosome loci. **(c)** Ratio of targeting of oRiboT over WT ribosomes with each level of intron overlap. ARF = allelic replacement frequency.

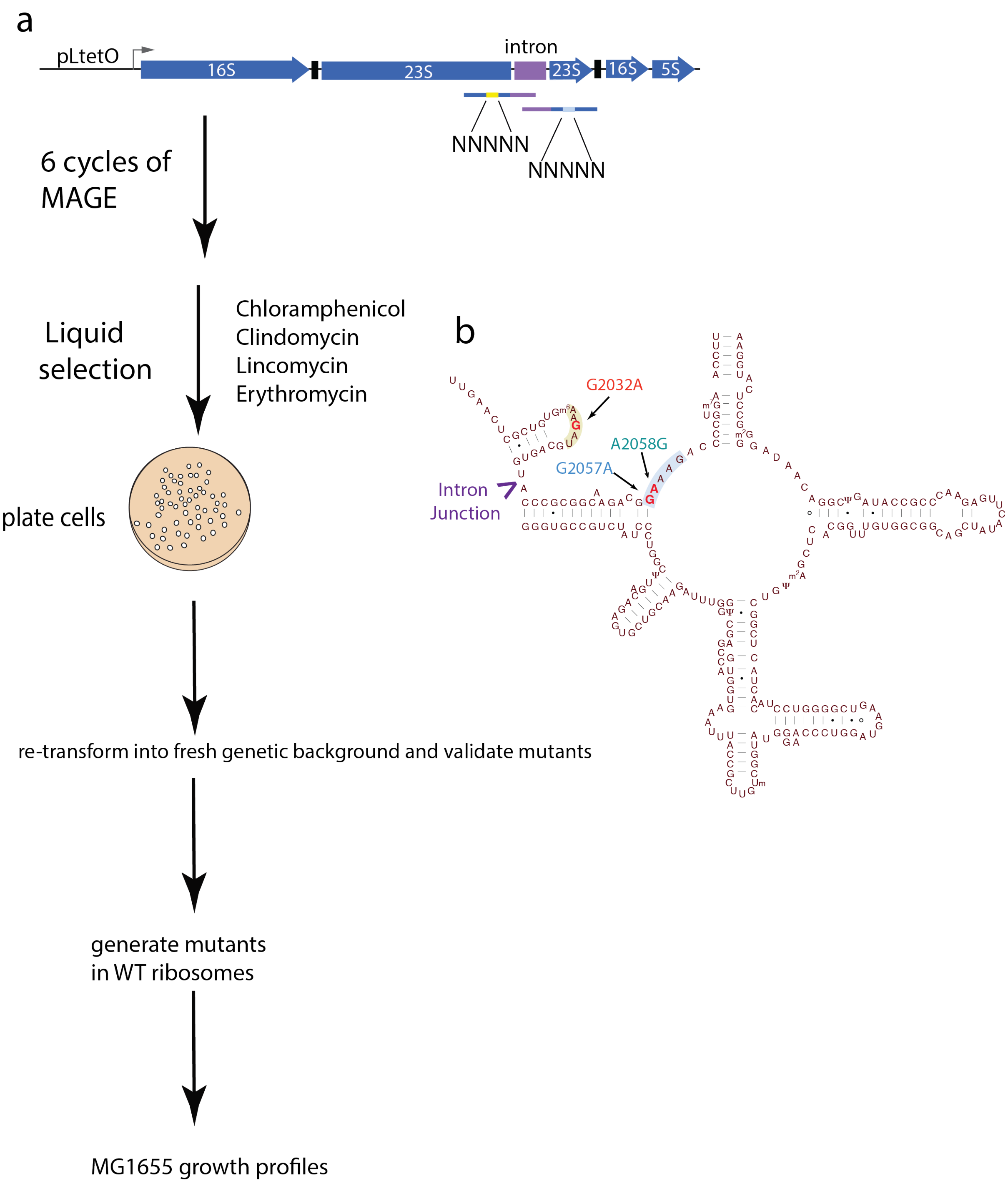

Supplementary Fig. 5. Mutagenesis and selection scheme used to evolve new ribosomal mutants with resistance to antibiotics targeting the large ribosomal subunit. (a) A ribosomal library was generated with f-MAGE using two MAGE ssODN pools that each contained five degenerate nucleotides (**Supplementary Table 2**) and which targeted regions 2030 - 2034 and 2057 - 2061, respectively. Antibiotic-resistant mutants were identified after liquid selections and validated in MAGE-enabled and WT strains. (b) The locations of the intron is indicated, as well as target sites for mutagenesis, oriented 5’ and 3’ to the intron-exon junction.

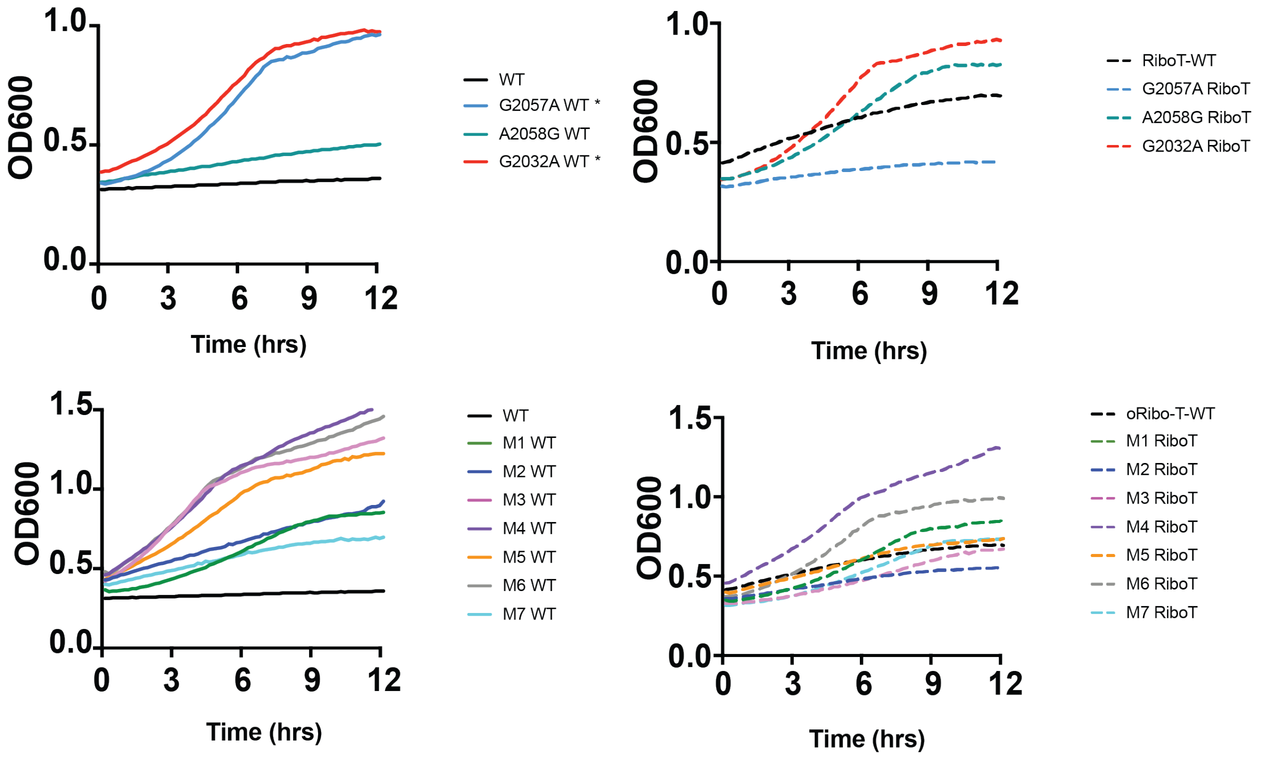

Supplementary Fig. 6. WT and oRiboT growth profiles in the presence of Chloramphenicol. MG1655 cells transformed with plasmids encoding WT RiboT or variants containing established (G2057A, A2058G, G2032A) or novel (M1-M7) mutations **(Table 1, Suplementary Table 4)** were grown in LB broth supplemented with chloramphenicol (7.74µM) for 12 h (n=3 biologically independent replicates). Mutations previously reported to confer antibiotic resistance are indicated with an asterisk (*).

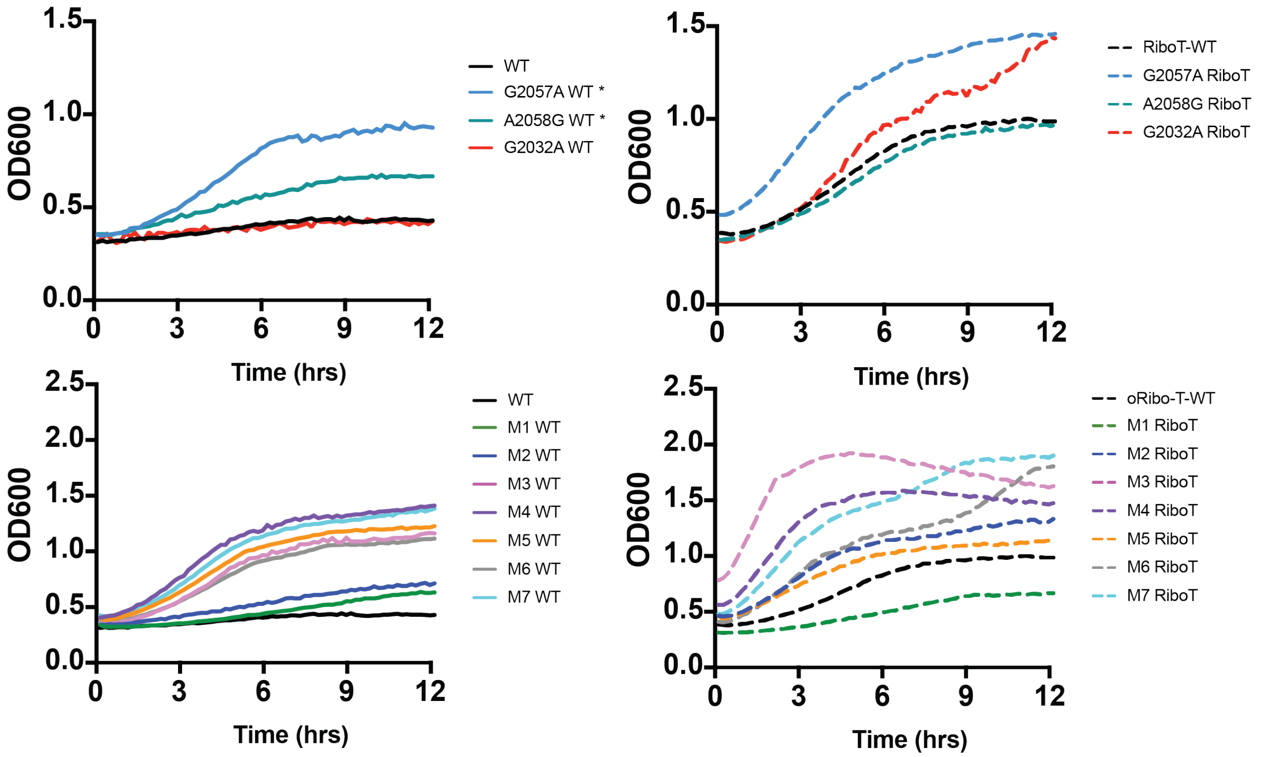

Supplementary Fig. 7. WT and oRiboT growth profiles in the presence of Erythromycin. MG1655 cells transformed with plasmids encoding WT RiboT or variants containing established (G2057A, A2058G, G2032A) or novel (M1-M7) mutations **(Table 1, Suplementary Table 4)** were grown in LB broth supplemented with erythromycin (273µM) for 12 h (n=3 biologically independent replicates). Mutations previously reported to confer antibiotic resistance are indicated with an asterisk (*).

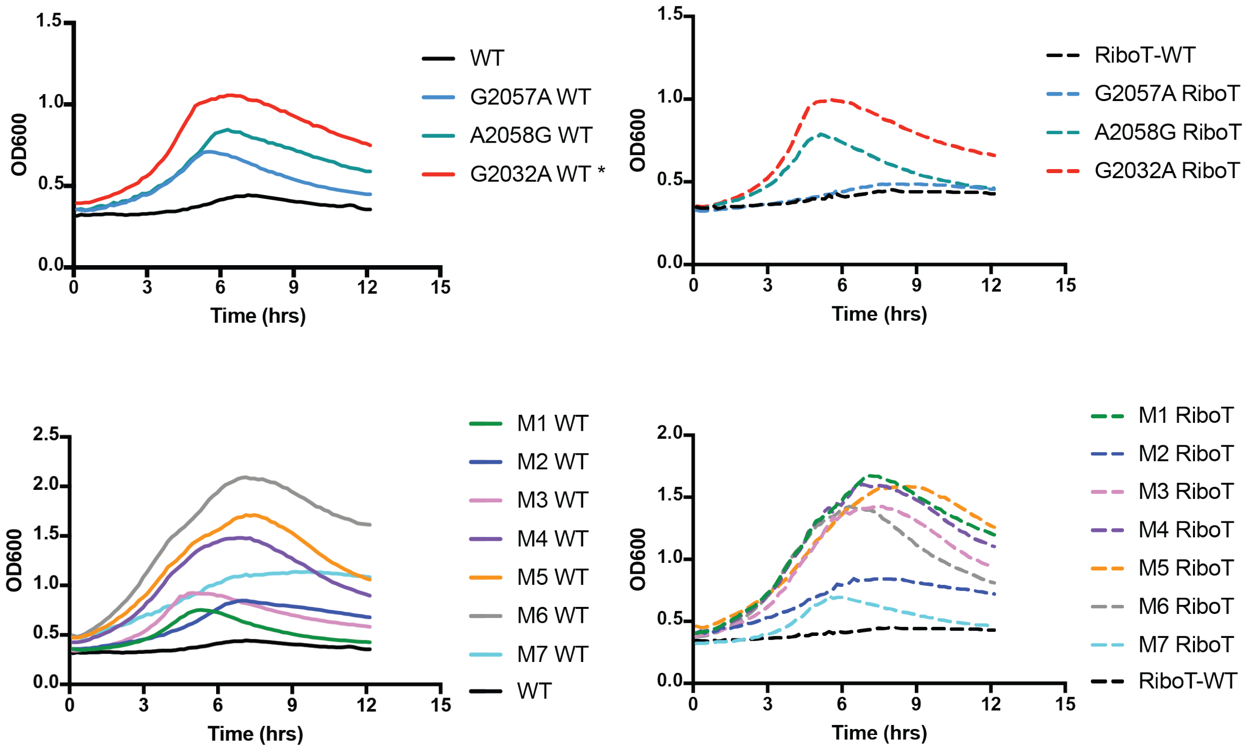

Supplementary Fig. 8. WT and oRiboT growth profiles in the presence of Clindamycin. MG1655 cells transformed with plasmids encoding WT RiboT or variants containing established (G2057A, A2058G, G2032A) or novel (M1-M7) mutations **(Table 1, Suplementary Table 4)** were grown in lincomycin (1.3 mM) for 12 h (n=3 biologically independent replicates). Mutations previously reported to confer antibiotic resistance are indicated with an asterisk (*).

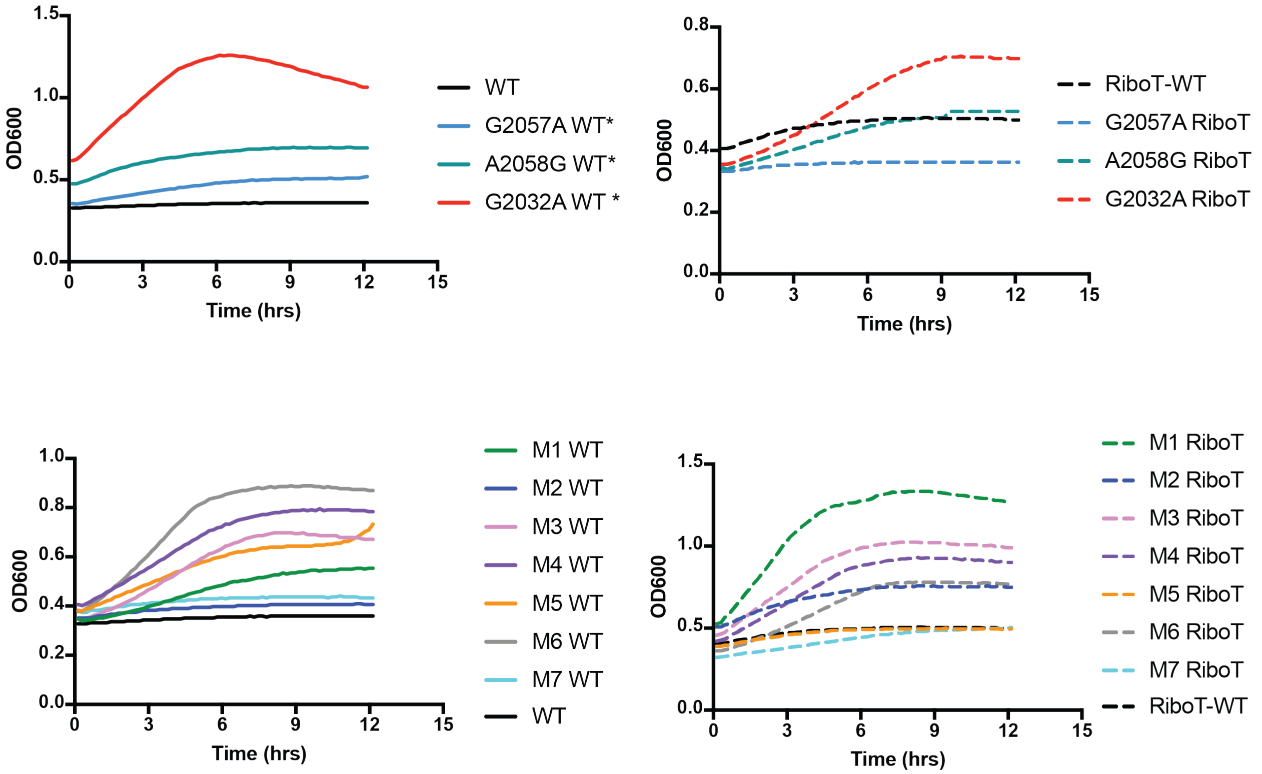

Supplementary Fig. 9. WT and oRiboT growth profiles in Lincomycin. MG1655 cells transformed with plasmids encoding WT RiboT or variants containing established (G2057A, A2058G, G2032A) or novel (M1-M7) **(Table 1, Supplementary Table 4)** mutations were grown in lincomycin (28.22 mM) for 12 h (n=3 biologically independent replicates). Mutations previously reported to confer antibiotic resistance are indicated with an asterisk (*).

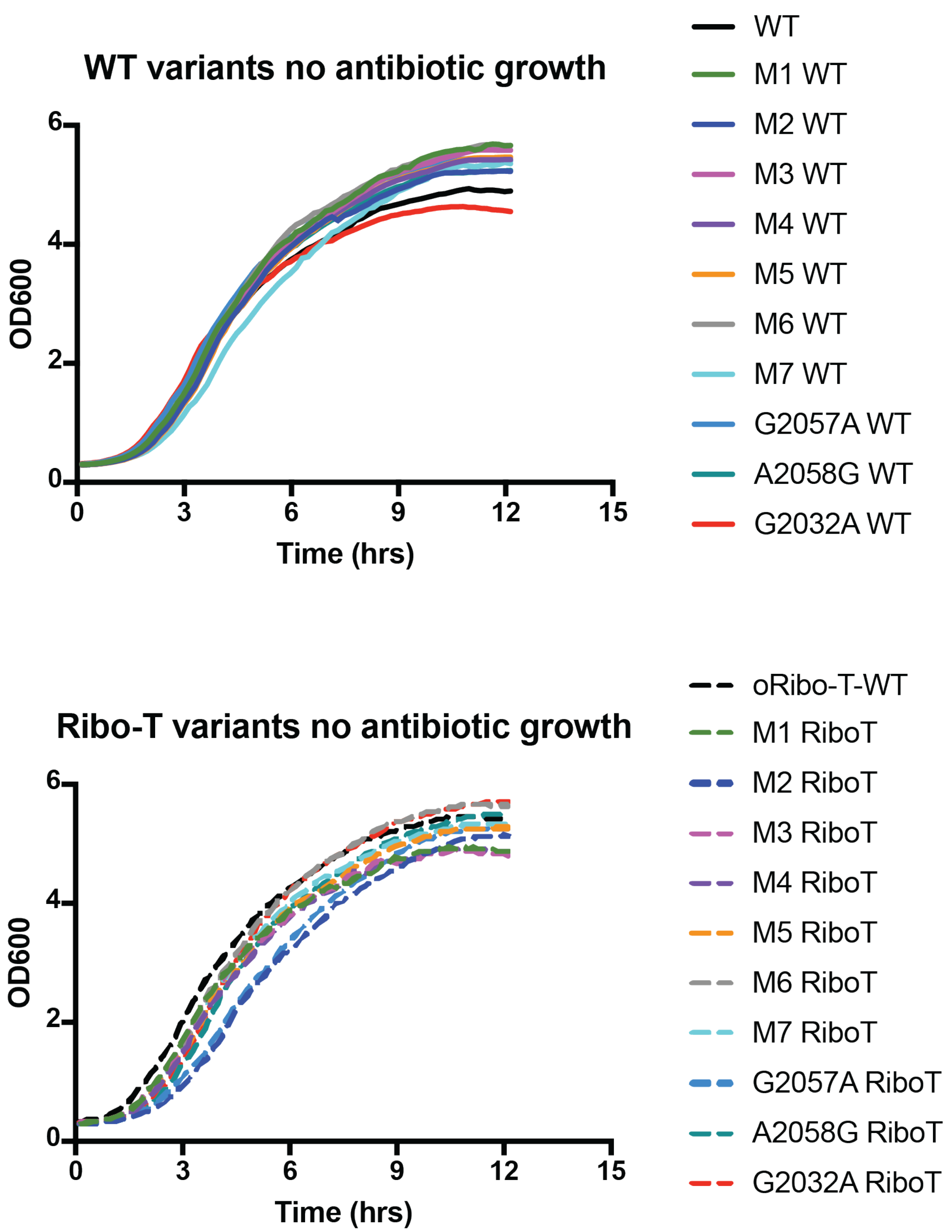

Supplementary Fig. 10. WT and oRiboT growth profiles without antibiotics. MG1655 cells were transformed with plasmids encoding WT or RiboT rRNA or variants thereof containing established (G2057A, A2058G, G2032A) or novel (M1-M7) mutations **(Table 1, Supplementary Table 4)** and growth monitored at 600 nm as a function of time.

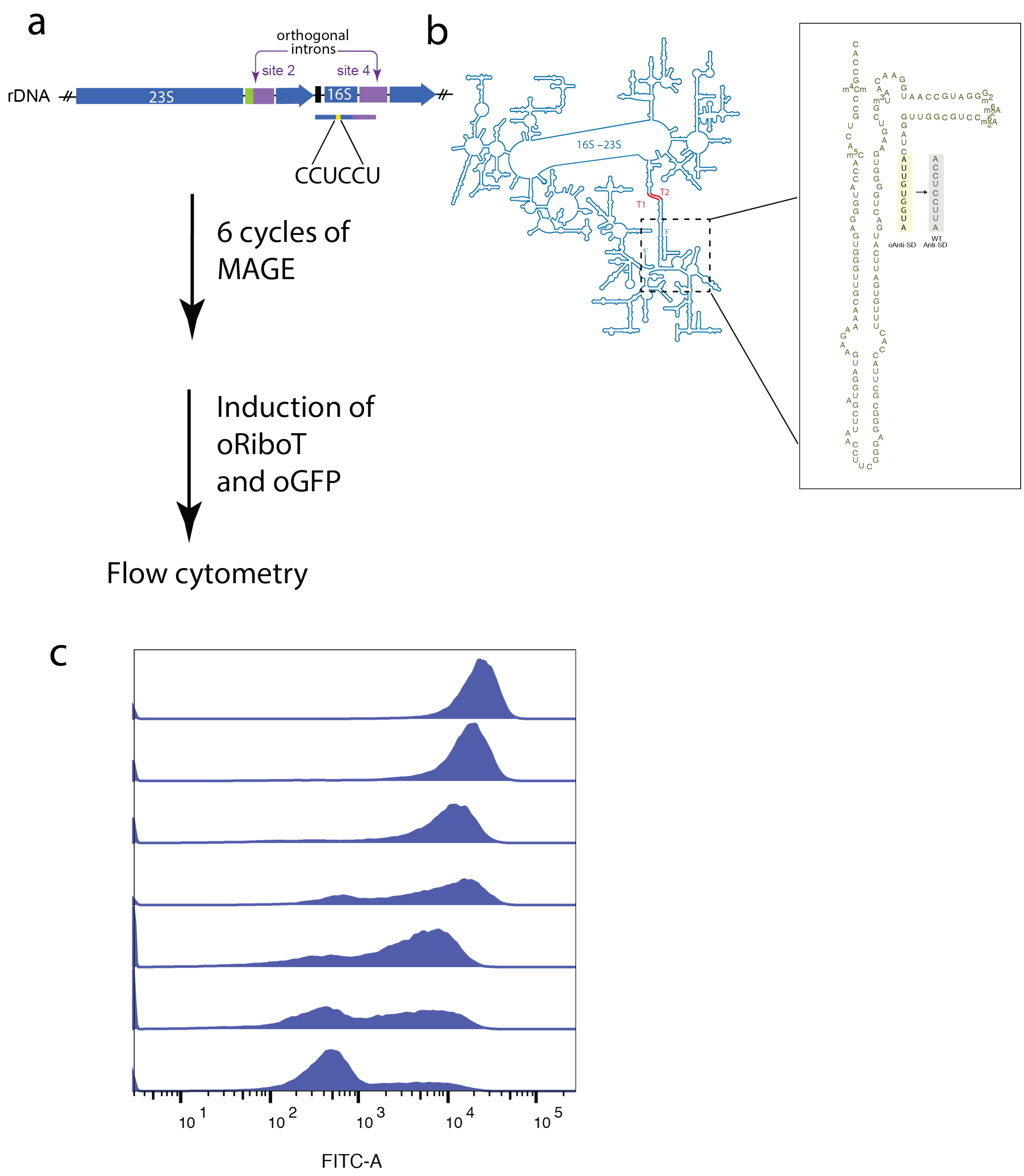

Supplementary Fig. 11. ***In vivo*** **ribosome mutagenesis with f-MAGE.** (a) f-MAGE performed to validate *in vivo* evolution at the aSD (site 4). Six cycles of f-MAGE with ssODN to convert the anti-oRBS to WT-anti-SD sequence were performed on C321 strain with oRiboT-CTt2-Tt4 and oGFP reporter. Post-MAGE cultures were induced for oGFP expression and flow cytometry was performed on cultures from f-MAGE cycles 0 - 6. (b) Location of orthogonal aSD is indicated and sequence after conversion by ssODN to WT aSD. (c) The oGFP fluorescence of populations induced after cycles 0 -6 of fMAGE was visualized with flow cytometry.

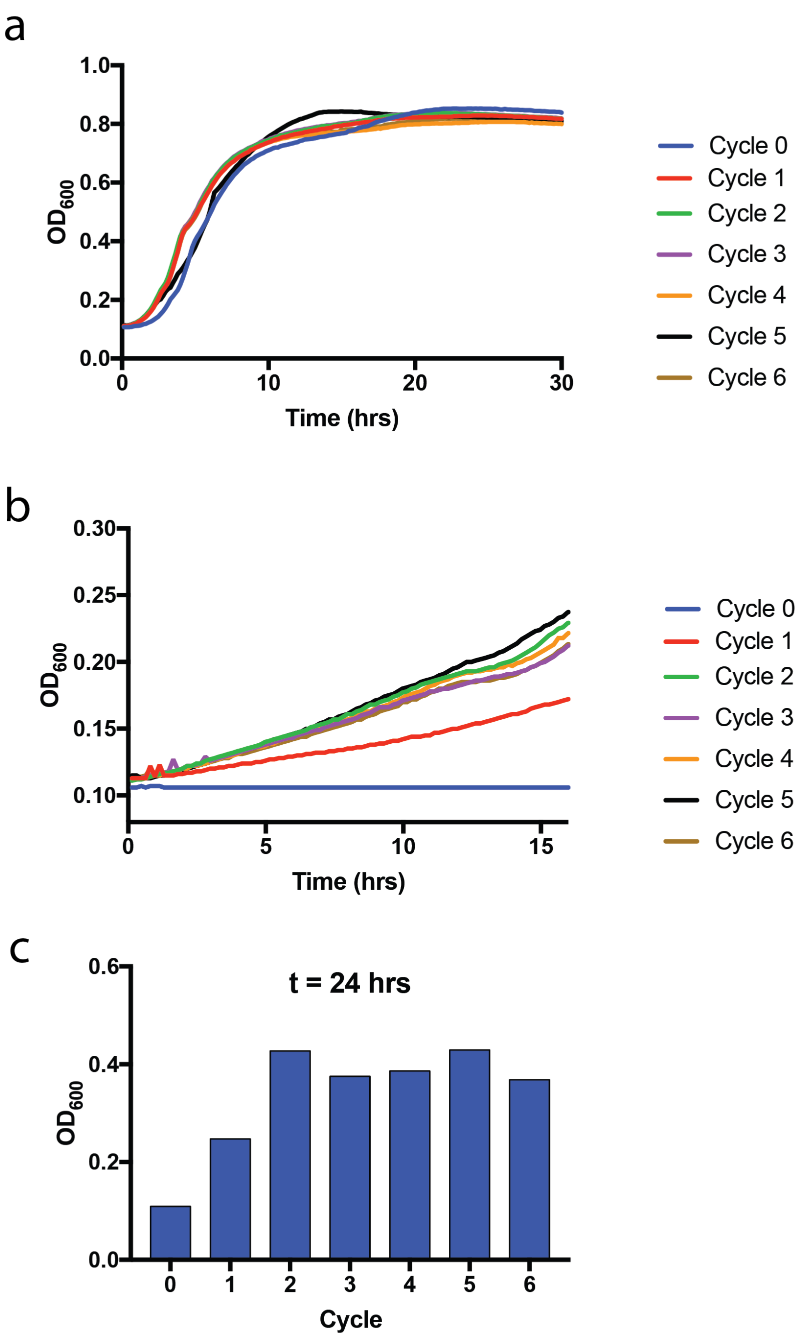

Supplementary Fig. 12. Growth profiles in chloramphenicol after multisite f-MAGE with two discrete ssODNs. f-MAGE was performed in *E. coli* C321 strain transformed with plasmids encoding oRiboT-CTt2-Tt4 and oGFP reporter with ssODN targeting site 2 to make M4 mutation **(Table 1)** and ssODN targeting site 4 to switch the orthogonal oSD to WT aSD. Cells recovered after each cycle of f-MAGE were grown in (a) LB, or (b) LB supplemented with chloramphenicol (7.74 μM), and growth was monitored at 600 nm as a function of time. (c) Endpoint OD_{600} measurements for each cycle were recorded at 24hrs.

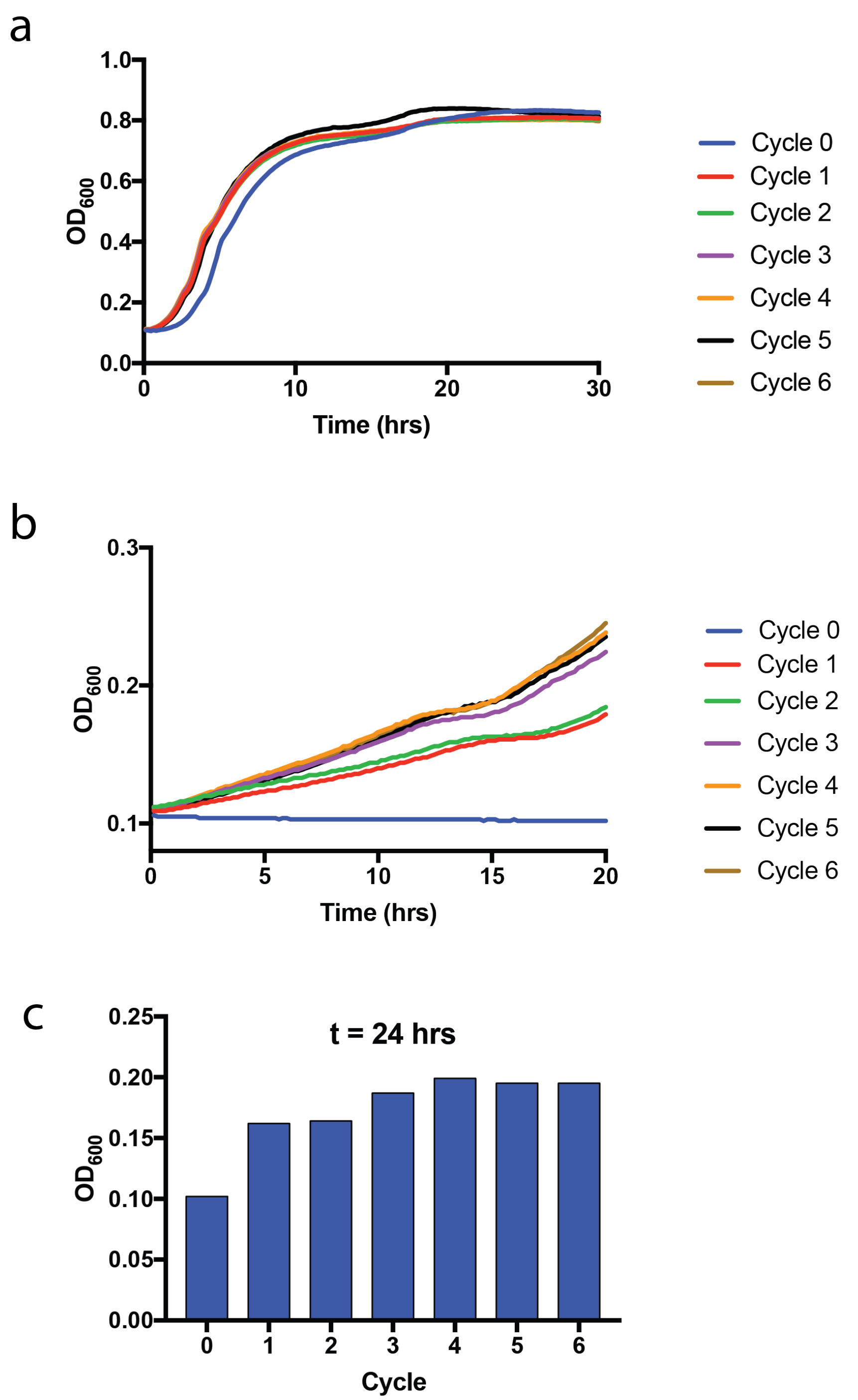

Supplementary Fig. 13. Growth profiles in chloramphenicol after multisite f-MAGE with a degenerate and discrete set of ssODNs. f-MAGE was performed in *E. coli* C321 strain transformed with plasmids encoding oRiboT-CTt2-Tt4 and oGFP reporter with ssODN targeting site 2 to make a degenerate library 5' and 3' to the intron, respectively **(Supplementary Fig. 5)** and ssODN targeting site 4 to switch the orthogonal oSD to WT aSD. Cells recovered after each cycle of f-MAGE were grown in (a) LB, or (b) LB supplemented with chloramphenicol (7.74 µM), and growth was monitored at 600 nm as a function of time. (c) Endpoint OD_{600} measurements for each cycle were recorded at 24hrs.

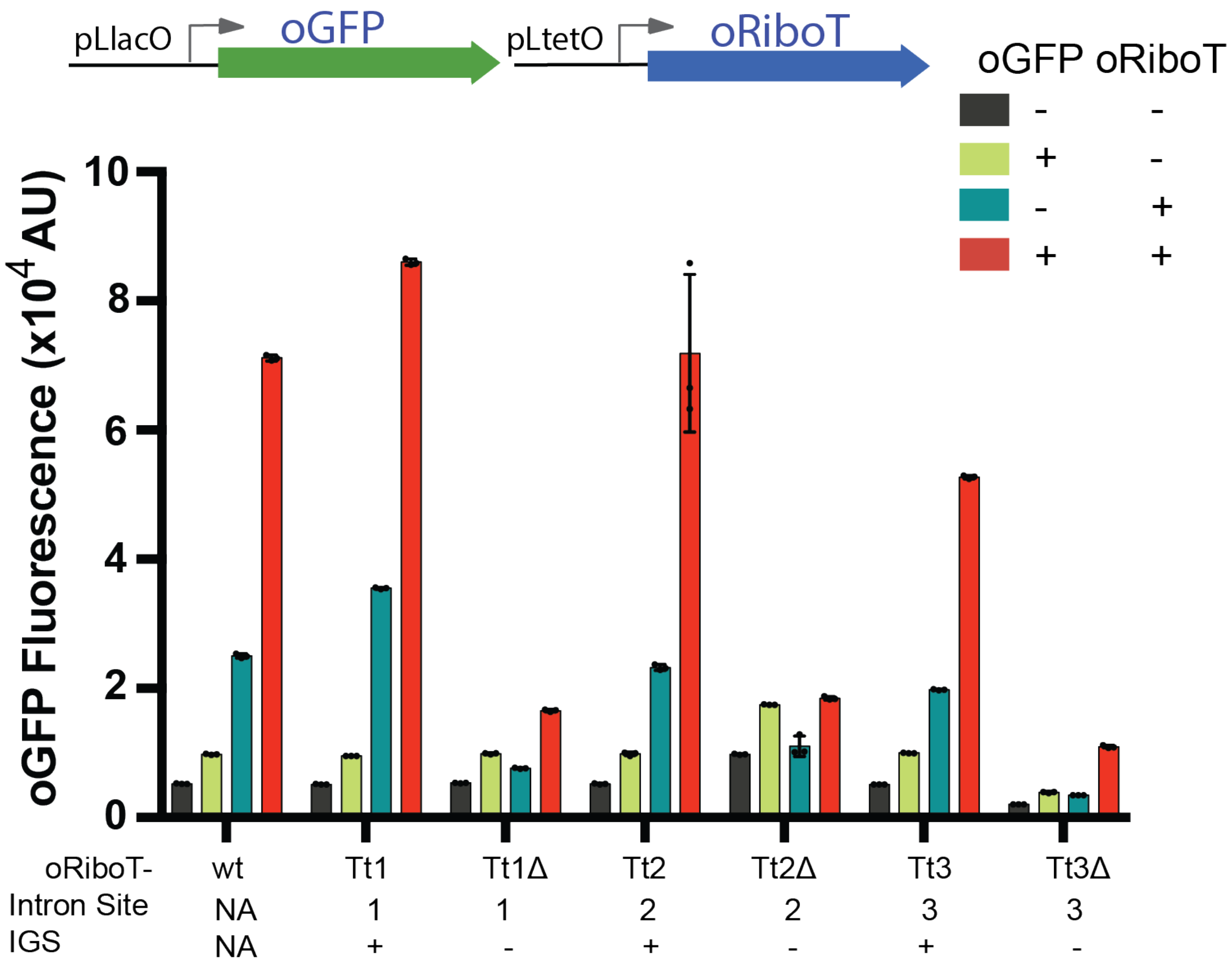

Supplementary Fig. 14. Measurement of oGFP expression by oRiboT and oRiboT intron variants. Expression of oGFP by WT oRiboT or variants whose genes contained functional (Tt1, Tt2, Tt3) or ablated (Tt1Δ, Tt2Δ, Tt3Δ) introns (*i.e,* internal guide sequence deletion, ΔIGS). Values and error bars represent the mean and standard deviation of n=3 biologically independent replicates(dots).

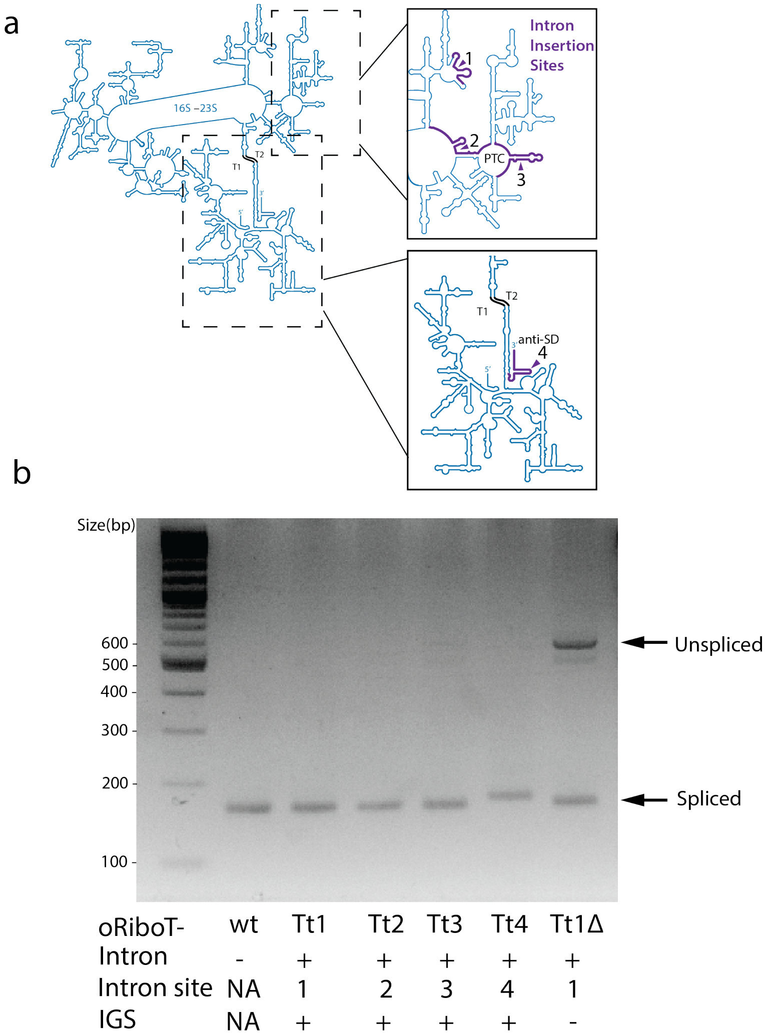

Supplementary Fig. 15. Validation of *in vivo* intron splicing at sites 1 - 4. (a) sites of insertion of Tt intron into oRiboT **(b)** RT-PCR was performed on total purified RNA from cells expressing oRiboT-Tt1, -Tt2, -Tt3, -Tt4 (+IGS) and oRiboT-Tt1Δ (ΔIGS) in order to determine whether introns were completely spliced out of oRiboT and ligated post-splicing. These are representative results from n=2 independent experiments.

Engineered CTt intron (site 2)

GAACATCAGTGCTACTGACGCACTGAAAAGTTATCAGGCATGCACCTGGTAGCTAGT
CTTTAAACCAATAGATTGCATCGGTTTAAAAGGCAAGACCGTCAAATTGCGGGAAA
GGGGTCAACAGCCGTTCAGTACCAAGTCTCAGGGGAAACTTTGAGATGGCCTTGCA
AAGGGTATGGTAATAAGCTGACGGACATGGTCCTAACCACGCAGCCAAGTCCTAAG
TCAACAGATCTTCTGTTGATATGGATGCAGTTCACAGACTAAATGTCGGTCGGGGAA
GATGTATTCTTCTCATAAGATATAGTCGGACCTCTCCTTAATGGGAGCTAGCGGATG
AAGTGATGCAACACTGGAGCCGCTGGGAACTAATTTGTATGCGAAAGTATATTGATT
AGTTTTGGAGTACTCG

Natural Tt intron (site 2)

AAATAGCAATATTTACCTTTGCACTGAAAAGTTATCAGGCATGCACCTGGTAGCTAG
TCTTTAAACCAATAGATTGCATCGGTTTAAAAGGCAAGACCGTCAAATTGCGGGAA
AGGGGTCAACAGCCGTTCAGTACCAAGTCTCAGGGGAAACTTTGAGATGGCCTTGC
AAAGGGTATGGTAATAAGCTGACGGACATGGTCCTAACCACGCAGCCAAGTCCTAA
GTCAACAGATCTTCTGTTGATATGGATGCAGTTCACAGACTAAATGTCGGTCGGGGA
AGATGTATTCTTCTCATAAGATATAGTCGGACCTCTCCTTAATGGGAGCTAGCGGAT
GAAGTGATGCAACACTGGAGCCGCTGGGAACTAATTTGTATGCGAAAGTATATTGA
TTAGTTTTGGAGTACTCG

Supplementary Fig. 16. Sequences of Engineered CTt intron and natural Tt intron. The P1 sequence is indicated in red and IGS indicated in cyan.

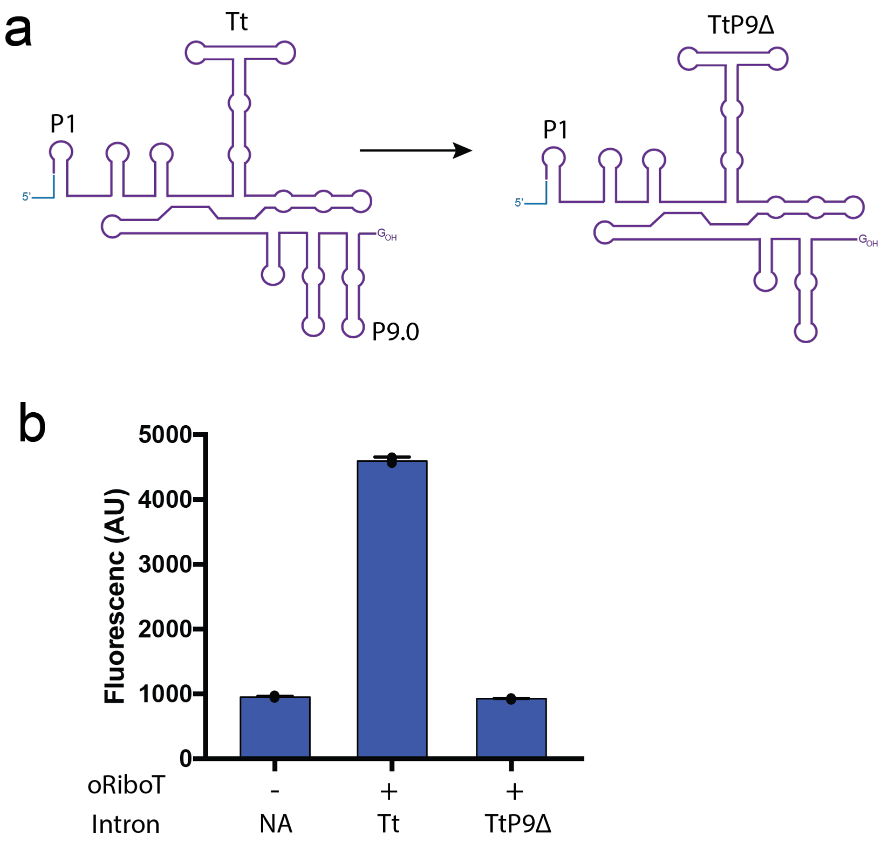

Supplementary Fig. 17. Construction and characterization of oRiboT variant with Tt intron with deletion of the P9.0 helix (TtP9Δ).

(a) Intron TtP9Δ was constructed by deleting the P9.0 helix from the Tt intron. It was inserted into oRiboT to create oRiboT-TtP9Δ in order to determine if this deletion would be tolerated in oRiboT function. **(b)** Expression of oGFP by cells containing no oRiboT, oRiboT-Tt2, or oRiboT-TtP9Δ. Values and error bars represent the mean and standard deviation of n=3 biologically independent replicates(dots).

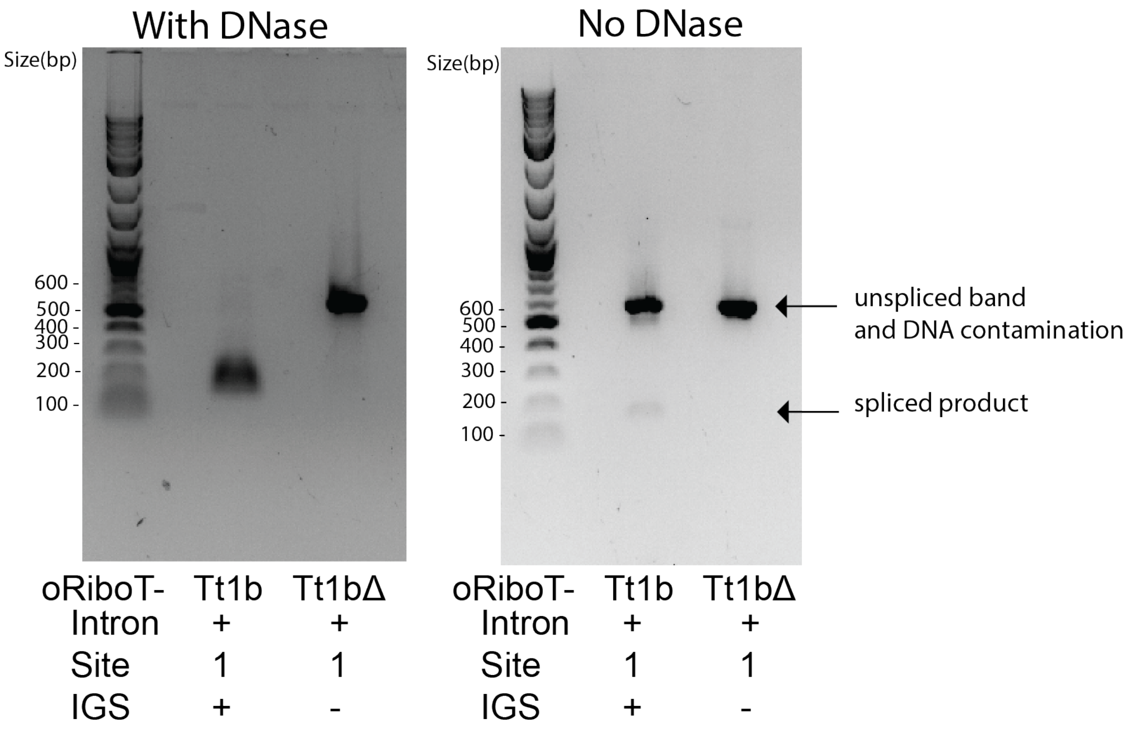

Supplementary Fig. 18. DNase and DNase-free controls for RT-PCR reactions to verify *in vivo* intron splicing. RT-PCR was performed using SuperScript OneStep RTPCR System with Platinum Taq DNA Polymerase on total purified RNA from oRiboT-Tti1b and oRiboT-Tti1bΔ. oRiboT-Tti1b and oRiboT-Tti1bΔ samples treated with DNase show the expected bands (left) whereas the same samples that had not been treated with DNase show an unspliced band suggesting genomic DNA contamination (right). Products of RT-PCR were analyzed by agarose gel electrophoresis. These are representative results from n=2 independent experiments.

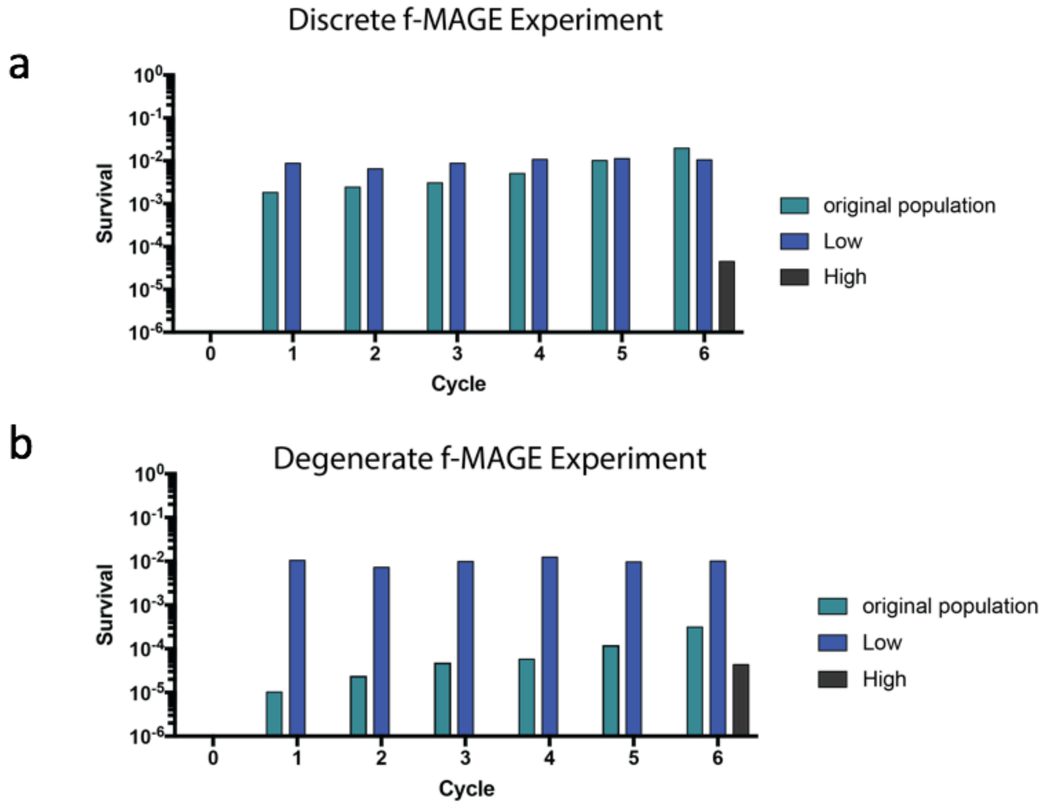

Supplementary Fig. 19. Quantification of surviving population members after low and high FACS sorts after oGFP induction. Populations of cycles 0-6 of fMAGE with discrete (a) or degenerate (b) ssODNs targeting site 2, along with ssODN switching oSD to WT were induced and sorted into low or high bins by oGFP fluorescence. The original population and low and high bins were grown after oRiboT induction on plates with 15.52μM chloramphenicol and aTc or no chloramphenicol and CFUs were normalized to calculate survival ratios. Each bar represents a single set of 15.52μM chloramphenicol and aTc, or no chloramphenicol, platings from individual sorts of $1x10^5$ cells.

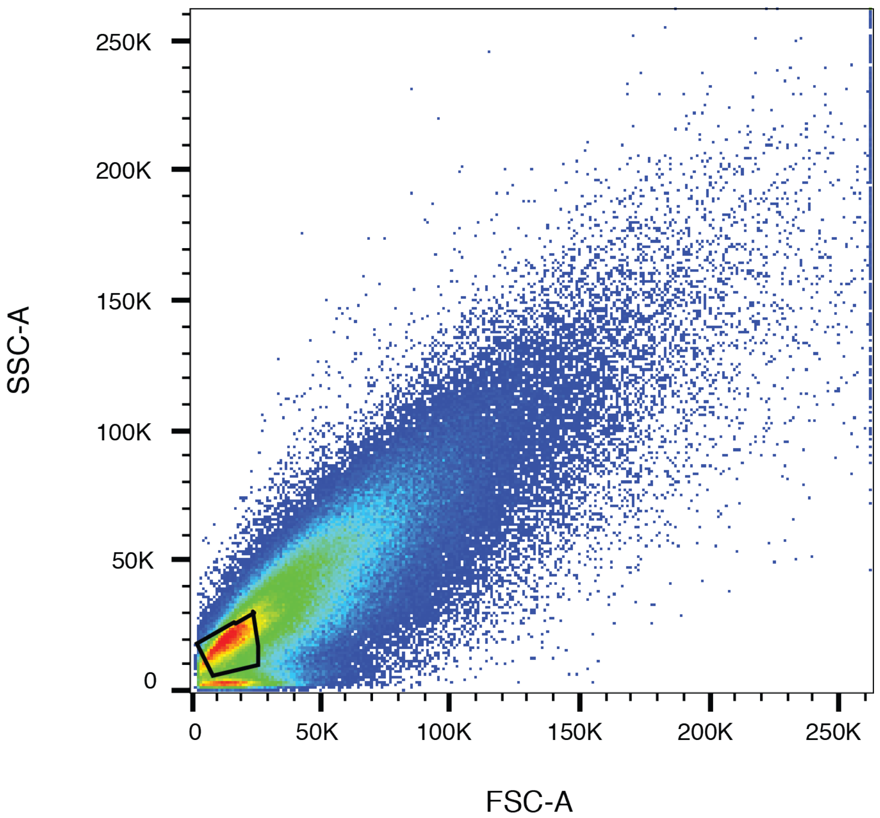

Supplementary Fig. 20. Gating strategy used for flow cytometry analysis of cell populations used in this study. A gate was drawn on the total population based on forward scatter (FSC-A) and side scatter (SSC-A) in order to analyze cells of similar size and morphology. The same gating strategy was employed for all samples.

Supplementary References

1. Ettayebi, M., Prasad, S.M. & Morgan, E.A. Chloramphenicol-erythromycin resistance mutations in a 23S rRNA gene of Escherichia coli. *Journal of Bacteriology* **162**, 551-557 (1985).
2. Douthwaite, S. Functional interactions within 23S rRNA involving the peptidyltransferase center. *Journal of bacteriology* **174**, 1333-1338 (1992).
3. Guo, F. & Cech, T.R. In vivo selection of better self-splicing introns in Escherichia coli: the role of the P1 extension helix of the Tetrahymena intron. *RNA* **8**, 647-658 (2002).
4. Beaudry, A.A. & Joyce, G.F. Minimum secondary structure requirements for catalytic activity of a self-splicing group I intron. *Biochemistry* **29**, 6534-6539 (1990).
5. Zarrinkar, P.P. & Williamson, J.R. The P9. 1–P9. 2 peripheral extension helps guide folding of the Tetrahymena ribozyme. *Nucleic acids research* **24**, 854-858 (1996).
6. Lutz, R. & Bujard, H. Independent and tight regulation of transcriptional units in Escherichia coli via the LacR/O, the TetR/O and AraC/I1-I2 regulatory elements. *Nucleic acids research* **25**, 1203-1210 (1997).
7. St-Pierre, F.o. et al. One-step cloning and chromosomal integration of DNA. *ACS synthetic biology* **2**, 537-541 (2013).
8. Meng, Q., Zhang, Y. & Liu, X.-Q. Rare group I intron with insertion sequence element in a bacterial ribonucleotide reductase gene. *Journal of bacteriology* **189**, 2150-2154 (2007).
9. Ko, M., Choi, H. & Park, C. Group I self-splicing intron in the recA gene of Bacillus anthracis. *Journal of bacteriology* **184**, 3917-3922 (2002).
10. Takashima, M. & Nakase, T. A phylogenetic analysis of three group I introns found in the nuclear small subunit ribosomal RNA gene of the ballistoconidiogenous anamorphic yeast-like fungus Tilletiopsis flava. *Genes & genetic systems* **72**, 205-214 (1997).
11. Bonocora, R.P. & Shub, D.A. A self-splicing group I intron in DNA polymerase genes of T7-like bacteriophages. *Journal of bacteriology* **186**, 8153-8155 (2004).
12. Tanner, M. & Cech, T. Activity and thermostability of the small self-splicing group I intron in the pre-tRNA (lle) of the purple bacterium Azoarcus. *Rna* **2**, 74-83 (1996).
13. Testa, S.M., Haidaris, C.G., Gigliotti, F. & Turner, D.H. A Pneumocystis carinii group I intron ribozyme that does not require 2 ‘OH groups on its 5 ‘exon mimic for binding to the catalytic core. *Biochemistry* **36**, 15303-15314 (1997).
14. Reinhold-Hurek, B. & Shub, D.A. Self-splicing introns in tRNA genes of widely divergent bacteria. *Nature* **357**, 173 (1992).
15. Michel, F. et al. Activation of the catalytic core of a group I intron by a remote 3'splice junction. *Genes & development* **6**, 1373-1385 (1992).
16. Komor, A.C., Badran, A.H. & Liu, D.R. CRISPR-based technologies for the manipulation of eukaryotic genomes. *Cell* **168**, 20-36 (2017).
17. Gaj, T., Gersbach, C.A. & Barbas III, C.F. ZFN, TALEN, and CRISPR/Cas-based methods for genome engineering. *Trends in biotechnology* **31**, 397-405 (2013).
18. Gaudelli, N.M. et al. Programmable base editing of A• T to G• C in genomic DNA without DNA cleavage. *Nature* **551**, 464-471 (2017).

19. Anzalone, A.V., Koblan, L.W. & Liu, D.R. Genome editing with CRISPR–Cas nucleases, base editors, transposases and prime editors. *Nature biotechnology* **38**, 824-844 (2020).
20. Anzalone, A.V. et al. Search-and-replace genome editing without double-strand breaks or donor DNA. *Nature* **576**, 149-157 (2019).
21. Wang, H.H. et al. Programming cells by multiplex genome engineering and accelerated evolution. *Nature* **460**, 894-898 (2009).
22. Urban, A., Neukirchen, S. & Jaeger, K.-E. A rapid and efficient method for site-directed mutagenesis using one-step overlap extension PCR. *Nucleic acids research* **25**, 2227-2228 (1997).
23. Cirino, P.C., Mayer, K.M. & Umeno, D. in Directed evolution library creation 3-9 (Springer, 2003).
24. Hogrefe, H.H., Cline, J., Youngblood, G.L. & Allen, R.M. Creating randomized amino acid libraries with the QuikChange® multi site-directed mutagenesis kit. *Biotechniques* **33**, 1158-1165 (2002).
25. Wang, W. & Malcolm, B.A. in In vitro mutagenesis protocols 37-43 (Springer, 2002).
26. Badran, A.H. & Liu, D.R. Development of potent in vivo mutagenesis plasmids with broad mutational spectra. *Nature communications* **6**, 1-10 (2015).
27. Halperin, S.O. et al. CRISPR-guided DNA polymerases enable diversification of all nucleotides in a tunable window. *Nature* (2018).
28. Esvelt, K.M., Carlson, J.C. & Liu, D.R. A system for the continuous directed evolution of biomolecules. *Nature* **472**, 499-503 (2011).
