# Supplementary material for: Targeted editing and evolution of engineered ribosomes in vivo by filtered editing
Source: Nat Commun. 2022 Jan 10;13:180. doi: 10.1038/s41467-021-27836-x (PMC8748908; doi:10.1038/s41467-021-27836-x)
Supplement: Supplementary file 2 — Reporting Summary [file 41467_2021_27836_MOESM2_ESM.pdf]

nature research

Corresponding author(s): Farren Isaacs

Last updated by author(s): November 18, 2021

Reporting Summary

Nature Research wishes to improve the reproducibility of the work that we publish. This form provides structure for consistency and transparency in reporting. For further information on Nature Research policies, see our Editorial Policies and the Editorial Policy Checklist.

Statistics

For all statistical analyses, confirm that the following items are present in the figure legend, table legend, main text, or Methods section.

n/a	Confirmed	
☐	☒	The exact sample size (n) for each experimental group/condition, given as a discrete number and unit of measurement
☐	☒	A statement on whether measurements were taken from distinct samples or whether the same sample was measured repeatedly
☒	☐	The statistical test(s) used AND whether they are one- or two-sided<br>*Only common tests should be described solely by name; describe more complex techniques in the Methods section.*
☒	☐	A description of all covariates tested
☒	☐	A description of any assumptions or corrections, such as tests of normality and adjustment for multiple comparisons
☐	☒	A full description of the statistical parameters including central tendency (e.g. means) or other basic estimates (e.g. regression coefficient) AND variation (e.g. standard deviation) or associated estimates of uncertainty (e.g. confidence intervals)
☒	☐	For null hypothesis testing, the test statistic (e.g. F, t, r) with confidence intervals, effect sizes, degrees of freedom and P value noted<br>*Give P values as exact values whenever suitable.*
☒	☐	For Bayesian analysis, information on the choice of priors and Markov chain Monte Carlo settings
☒	☐	For hierarchical and complex designs, identification of the appropriate level for tests and full reporting of outcomes
☐	☐	Estimates of effect sizes (e.g. Cohen's d, Pearson's r), indicating how they were calculated

Our web collection on statistics for biologists contains articles on many of the points above.

Software and code

Policy information about availability of computer code

Data collection	BD FACSDiva software v.8 was used for flow cytometry data collection.
Data analysis	Python 2.7 was used for high throughput sequencing data analysis. FlowJo software was used for flow cytometry data analysis.

For manuscripts utilizing custom algorithms or software that are central to the research but not yet described in published literature, software must be made available to editors and reviewers. We strongly encourage code deposition in a community repository (e.g. GitHub). See the Nature Research guidelines for submitting code & software for further information.

Data

Policy information about availability of data

All manuscripts must include a data availability statement. This statement should provide the following information, where applicable:

- Accession codes, unique identifiers, or web links for publicly available datasets
- A list of figures that have associated raw data
- A description of any restrictions on data availability

All data generated or analyzed during this study are included in this published article (and supplementary information). The original raw NGS data from f-MAGE and f-CRISPR quantification experiments can be found at: https://figshare.com/projects/Filtered_Editing/126737. Source data are provided with this paper.

Field-specific reporting

Please select the one below that is the best fit for your research. If you are not sure, read the appropriate sections before making your selection.

☒ Life sciences ☐ Behavioural & social sciences ☐ Ecological, evolutionary & environmental sciences

For a reference copy of the document with all sections, see nature.com/documents/nr-reporting-summary-flat.pdf

Life sciences study design

All studies must disclose on these points even when the disclosure is negative.

Sample size	A sample size of 3 was chosen for growth curve analysis and fluorescence measurements, with a minimum of 3 biological replicates for each condition. These sample sizes were determined based on literature precedent for these experimental conditions; please see: Amiram et al. 2015 (https://doi.org/10.1038/nbt.3372) and Orelle et al. 2015 (https://doi.org/10.1038/nature14862).
Data exclusions	There were no data exclusions.
Replication	We repeated all experiments with n=3. All attempts at replication were successful.
Randomization	Randomized mutations were made with f-MAGE in the PTC/Exit Tunnel of the ribosome for antibiotic evolution experiments. In all other experiments, samples were treated under identical conditions, so randomization was not used.
Blinding	All colony counts for antibiotic selection experiments were performed blindly, without knowledge of experimental conditions in each sample. For all other experiments, samples were treated identically, so blinding was not used.

Reporting for specific materials, systems and methods

We require information from authors about some types of materials, experimental systems and methods used in many studies. Here, indicate whether each material, system or method listed is relevant to your study. If you are not sure if a list item applies to your research, read the appropriate section before selecting a response.

Materials & experimental systems

n/a	Involved in the study	
☒	☐	Antibodies
☒	☐	Eukaryotic cell lines
☒	☐	Palaeontology and archaeology
☒	☐	Animals and other organisms
☒	☐	Human research participants
☒	☐	Clinical data
☒	☐	Dual use research of concern

Methods

n/a	Involved in the study	
☒	☐	ChIP-seq
☐	☒	Flow cytometry
☒	☐	MRI-based neuroimaging

Flow Cytometry

Plots

Confirm that:

☒ The axis labels state the marker and fluorochrome used (e.g. CD4-FITC).

☒ The axis scales are clearly visible. Include numbers along axes only for bottom left plot of group (a 'group' is an analysis of identical markers).

☒ All plots are contour plots with outliers or pseudocolor plots.

☒ A numerical value for number of cells or percentage (with statistics) is provided.

Methodology

Sample preparation	Cells were resuspended in 1X PBS prior to flow cytometry
Instrument	BD FACS Aria
Software	BD FACSDiva

Cell population abundance	The purity was determined by plating the sorted fractions and confirming GFP was being expressed and that cells retained resistance or sensitivity to chloramphenicol, as relevant to each sample condition.
Gating strategy	We used the bulk cell population

☒ Tick this box to confirm that a figure exemplifying the gating strategy is provided in the Supplementary Information.
